# Impact of Duration of Neoadjuvant Aromatase Inhibitors on Molecular Expression Profiles in Estrogen Receptor–positive Breast Cancers

**DOI:** 10.1158/1078-0432.CCR-21-2718

**Published:** 2022-03-14

**Authors:** Milana A. Bergamino, Gabriele Morani, Joel Parker, Eugene F. Schuster, Mariana F. Leal, Elena López-Knowles, Holly Tovey, Judith M. Bliss, John F.R. Robertson, Ian E. Smith, Mitch Dowsett, Maggie C.U. Cheang

**Affiliations:** 1 Clinical Trials and Statistics Unit (ICR-CTSU)- Division of Clinical Studies, The Institute of Cancer Research, London, United Kingdom.; 2 Department of Genetics, University of North Carolina at Chapel Hill, Chapel Hill, North Carolina.; 3 Royal Marsden Hospital, London, United Kingdom.; 4 Faculty of Medicine & Health Sciences, Queen's Medical Centre, Nottingham, United Kingdom.; 5 Breast Cancer Now Research Centre, The Institute of Cancer Research, Sutton, London, United Kingdom.

## Abstract

**Purpose:**

Aromatase inhibitor (AI) treatment is the standard of care for postmenopausal women with primary estrogen receptor–positive breast cancer. The impact of duration of neoadjuvant endocrine therapy (NET) on molecular characteristics is still unknown. We evaluated and compared changes of gene expression profiles under short-term (2-week) versus longer-term neoadjuvant AIs.

**Experimental Design:**

Global gene expression profiles from the PeriOperative Endocrine Therapy for Individualised Care (POETIC) trial (137 received 2 weeks of AIs and 47 received no treatment) and targeted gene expression from 80 patients with breast cancer treated with NET for more than 1 month (NeoAI) were assessed. Intrinsic subtyping, module scores covering different cancer pathways and immune-related genes were calculated for pretreated and posttreated tumors.

**Results:**

The differences in intrinsic subtypes after NET were comparable between the two cohorts, with most Luminal B (90.0% in the POETIC trial and 76.3% in NeoAI) and 50.0% of HER2 enriched at baseline reclassified as Luminal A or normal-like after NET. Downregulation of proliferative-related pathways was observed after 2 weeks of AIs. However, more changes in genes from cancer-signaling pathways such as *MAPK* and *PI3K/AKT/mTOR* and immune response/immune-checkpoint components that were associated with AI-resistant tumors and differential outcome were observed in the NeoAI study.

**Conclusions:**

Tumor transcriptional profiles undergo bigger changes in response to longer NET. Changes in HER2-enriched and Luminal B subtypes are similar between the two cohorts, thus AI-sensitive intrinsic subtype tumors associated with good survival might be identified after 2 weeks of AI. The changes of immune-checkpoint component expression in early AI resistance and its impact on survival outcome warrants careful investigation in clinical trials.

**Translational Relevance**

Our study shows that neoadjuvant treatment with short- and longer-term aromatase inhibitors (AIs) in primary estrogen receptor-positive (ER^+^) breast cancer exerts comparable impact on changes in intrinsic subtypes between baseline and surgery. However, neoadjuvant AI treatment beyond 2 weeks leads more changes in molecular characteristics at a transcriptional level, such as genes involved in pathways like *MAPK* and *PI3K/AKT/mTOR* and characteristics for immune response landscape, including those covering immune-checkpoint component. These findings provide rationale for considering neoadjuvant AI therapy beyond 2 weeks in patients with high-risk ER^+^ breast cancer tumors. The role of immune-checkpoint component inhibition for endocrine therapy–resistant ER^+^ tumors in this setting warrants careful investigation.

## Introduction

Breast cancer is molecularly and clinically heterogeneous, and approximately 60% to 80% of cases are estrogen receptor–positive (ER^+^). The standard of care for postmenopausal women with ER^+^ breast cancer includes aromatase inhibitors (AIs) over a 5- to 10-year period. However, 20% to 25% of patients with ER^+^ breast cancer will eventually relapse, and additional biomarkers to identify resistance mechanisms to AIs are warranted (1–4).

Global gene expression analyses in breast cancer have shown molecular heterogeneity with a far more complex portrait beyond clinicopathologic classification (5–7). The elucidation of the molecular intrinsic subtypes has led to the categorization of breast cancer tumors into clinically relevant but molecular distinct subgroups that can be optimally defined by the 50 gene–based PAM50 classifier (8–10). These molecular subtypes are associated with different incidence and racial disparity, response to treatment and prognosis (8). However, there are still insufficient data about changes of those molecular characteristics under different lengths of AI treatment and whether pretreatment or posttreatment characteristics are better predictors of prognosis (11, 12).

Preoperative and neoadjuvant trials involving the collection of viable paired biopsies at diagnosis and at surgery provide a valuable source to understand genes and pathways involved in resistance to therapy, with the possibility to use, for example, Ki67 proliferation markers as a valuable endpoint associated with prognosis (13, 14). Our group previously suggested that reduced ER dependence and E2F-signaling activation after short- and long-term neoadjuvant AIs are associated with poor response (15, 16). However, we also reported the enrichment of *ESR1* mutation with long-term neoadjuvant AI in primary breast cancer using a real-world cohort of patients treated in the Royal Marsden Hospital (RMH; London, United Kingdom; ref. 16). Therefore, the comparison of the effect of different lengths of neoadjuvant AI therapy in molecular features might be necessary to elucidate the full impact on molecular alterations that might limit response and lead to clinical resistance.

In this study, the impact of short- and long-term neoadjuvant AI therapy on molecular changes, including intrinsic subtypes and signaling pathways was comprehensively evaluated. Gene expression profiles from two cohorts of patients with early primary ER^+^ breast cancer were analyzed: (i) the PeriOperative Endocrine Therapy for Individualised Care (POETIC) trial, in which patients were treated for 2 weeks (15) and (ii) patients treated for more than 1 month, named in the current study as NeoAI (16).

## Materials and Methods

### Patients' populations

Data from two different cohorts of postmenopausal women with primary ER^+^ breast cancer treated with different lengths of neoadjuvant AI were analyzed (Supplementary Fig. S1).

#### POETIC subset

The POETIC trial was a phase III, randomized study of 4,486 postmenopausal patients with ER^+^ breast cancer. Patients were randomized 2:1 to receive 2 weeks of preoperative AIs (letrozole 2.5 mg, anastrozole 1 mg per day orally) versus no treatment to determine whether perioperative AIs followed by standard adjuvant therapy would improve survival (15). The subset used in this study comprised 184 patients with paired samples: 137 tumors treated with AIs [86.1% (118) were human epidermal growth factors receptor not amplified or overexpressed (HER2^−^) and 13.9% (19) HER2^+^] and 47 patients who did not receive perioperative AIs as a control group.

#### NeoAI study

This was a retrospective cohort of patients treated with neoadjuvant AIs (letrozole 2.5 mg, anastrozole 1 mg or exemestane 25 mg per day orally) for at least 1 month (mean, 6.24 months ± SD, 3.9) at the RMH between 2003 and 2016 (16). Data from 80 patients from this study were analyzed: 93.8% (75) were HER2^−^ and 6.2% (5) HER2^+^. Seven patients with baseline Ki67% < 5% or lack of clinical data or gene expression were excluded. To provide a view of real-world AI-resistance mechanisms in ER^+^ breast cancer, both HER2^+^ and HER2^−^ were included in this study, with subsequent subgroup analyses focused on ER^+^ HER2^−^ tumors.

### Gene expression profiles

In the POETIC subset, gene expression data from microarray were obtained as described previously (15). Probes targeting 16,528 expressed genes (detection *P* < 0.01 in at least 20% of samples) were included in this analysis. Expression data were then log_2_ transformed and quantile normalized for downstream analysis, and probes were collapsed to gene-level expression based on the highest SD across samples. Expression levels of 649 published modules covering different cancer, immune response, and proliferation-related pathways were generated by taking the median of the genes available within the normalized microarray data (17).

In the NeoAI study, normalized log_2_ expression of 744 different genes covering the most important aspects of breast cancer—such as proliferation, invasion, *PI3K-AKT-mTOR* pathways, *MAPK* signaling, inflammation and the PAM50 gene set—previously analyzed using NanoString technology, were included (16, 18). We also explored the changes in two immune-related pathway module scores that had previously been reported to be associated with AI resistance and to predict benefit from immunotherapy (19, 20).

### PAM50 intrinsic subtypes

In the POETIC subset, each tumor sample was classified into one of the five intrinsic subtypes, namely Luminal A, Luminal B, Her2 enriched (Her2-E), basal-l, ike and normal-like using the 50-gene PAM50 classifier after subgroup-specific centering as reported previously (7, 15).

In the NeoAI study, the 46 genes raw expression values used in Prosigna were first normalized to eight housekeeping genes (*ACTB*, *GUS*, *MRPL19*, *PSMC4*, *PUM1*, *RPLP0*, *SF3A1*, and *TFRC*) and then normalized to a cohort of 229 sample ER^+^/HER2^−^ tumors previously subjected to the Prosigna assay for subgroup-median centering. Samples were finally classified using the PAM50 classifier applying the proper technical calibration factor as reported previously (21).

### Biomarker analysis

ER status was measured locally and centrally reviewed by IHC. HER2 status was measured locally using IHC and/or ISH. Ki67 proliferation rate was obtained by IHC from staining on formalin-fixed samples using anti–MIB-1 (M7240, DAKO UK). Ki67 rate was categorized into High (≥10%) and Low (<10%) at baseline and surgery. Tumors were also classified into four classes according to Ki67 changes between the two time points: High_baseline_-High_surgery_ (H-H), High_baseline_-Low_surgery_ (H-L), Low_baseline_-Low_surgery_ (L-L), and Low_baseline_-High_surgery_ (L-H) as reported previously (22).

### Statistical and data analysis

Statistical analysis was performed with R version 3.6.3 software. A two-tailed *P* value of less than 0.05 was considered statistically significant. *t* tests were applied in all unpaired comparisons. Paired *t* tests followed by Benjamini–Hochberg corrections for multiple comparisons were carried out to compare the changes of PAM50 intrinsic subtype's correlation scores between baseline and surgery biopsies. For module score, a combined threshold of significance was defined as *P*_adjusted_ < 0.05 and log_2_ fold change (FC) > |0.3785116|. For the single-gene analysis, a more restrictive fold-change threshold was applied (*log*_2_FC > |1|). Spearman rank correlation was used to explore the correlation of changes in intrinsic subtype classification, expression of some particular genes and/or module scores with duration of AI treatment in the NeoAI study. Significance Analysis of Microarrays (SAM analysis) was used to select key gene module scores associated with early AI resistance to evaluate the impact of AI on changes in their expression (23, 24). Survival analyses of time to recurrence (TTR) and overall survival (OS) in the POETIC and of OS in the NeoAI were performed respectively. Because of the lack of data of recurrence, within the NeoAI study, we also determined the association of changes in gene expression with risk of recurrence score (ROR score) at surgery as a surrogate biomarker of relapse. To do so, we previously assessed the correlation between ROR score at surgery and TTR in the POETIC subset (*P* 0.03). Multivariable Cox regression models adjusted for standard clinicopathologic variables including PR, HER2 status, tumor grade, pathologic tumor size, histologic type, nodal status, and vascular invasion were performed to assess the independent prognostic value of changes in gene expression and intrinsic subtypes.

### Ethics statement

The POETIC trial was approved by the London–South East Research Ethics Committee (reference 08/H1102/37). For the NeoAI study, ethical approval was received from an NHS research ethics committee (reference 17/EM/0145). Both studies were adopted by the Declaration of Helsinki and patients from both studies provided written informed consent to molecular analysis of their samples for research purposes.

### Data availability

Gene expression data from POETIC study can be found at Gene Expression Omnibus with the accession number: GSE126870. Additional data are available upon request by contacting the corresponding author or poetic-icrctsu@icr.ac.uk.

## Results

### Changes of intrinsic subtypes induced by short- and longer-term neoadjuvant AI therapy

The demographics and molecular characteristics of the patients of the two cohorts are shown in Supplementary Table S1. Baseline molecular characteristics were different between POETIC and NeoAI cohorts with the majority of samples being Luminal A (88/137; 64.2%) in the POETIC-treated samples subset and Luminal B (38/121; 47.5%) in NeoAI. The rest of the baseline clinicopathologic characteristics were similar among the two subsets.

**Figure 1.**
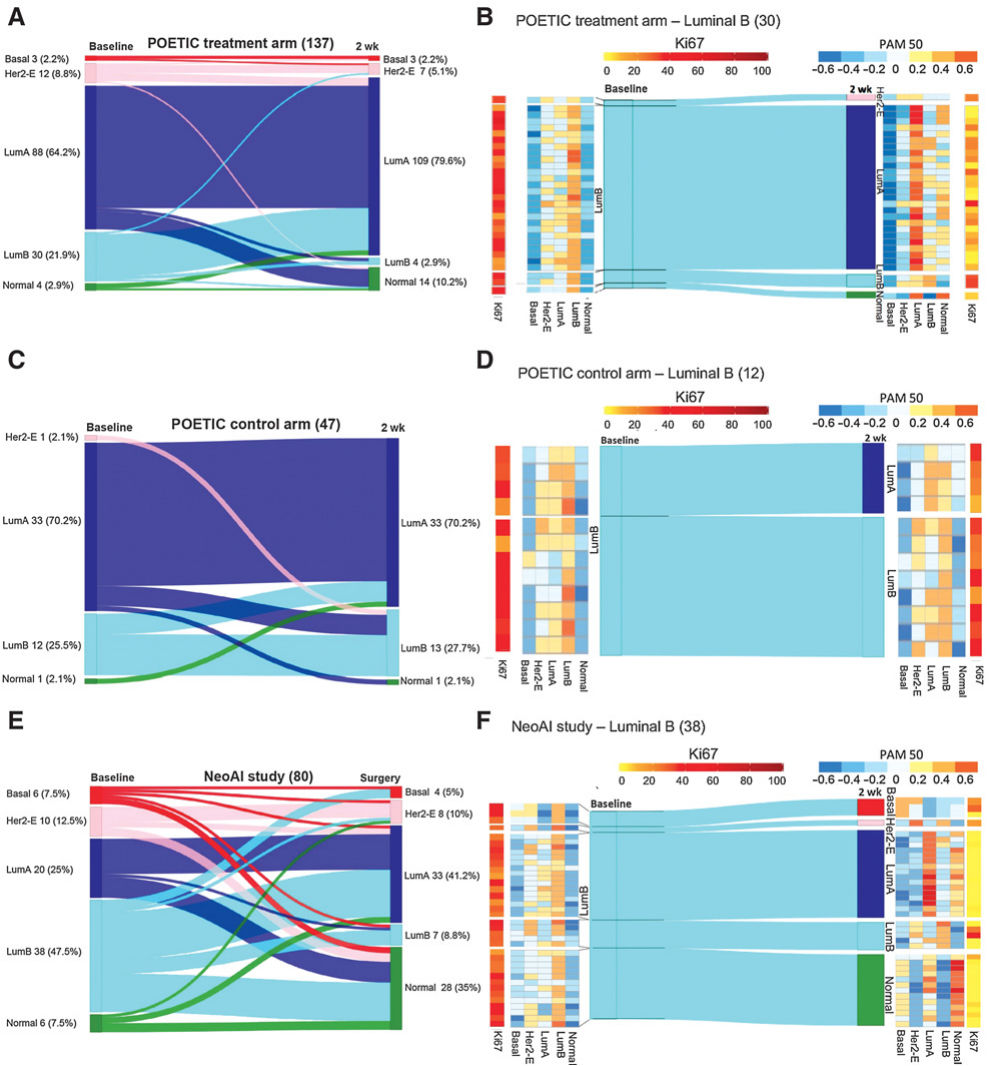
Differences in intrinsic subtype classification from baseline to surgery in the POETIC subset and the NeoAI study. Changes of intrinsic subtype classifications in all the POETIC-treated samples (**A**); in POETIC Luminal B–treated samples (**B**); in POETIC control samples (**C**); in POETIC Luminal B control samples (**D**); in all the NeoAI study samples (**E**), and in NeoAI Luminal B samples (**F**). Her2-E, Her2 enriched; LumB, Luminal B; LumA, Luminal A; 2 wk, 2-week time point.

The differences of intrinsic subtype between baseline and surgery were more frequent in the POETIC treatment group than in the controls (38% vs. 23.4%). In the treated group, most Luminal B tumors at baseline (90.0%, 27/30) and 50.0% (6/12) of Her2-E were redesignated as Luminal A or normal-like subtypes (Fig. 1A and B), whereas 41.7% of Her2-E and most Luminal A, 85.2% (75/88) and basal-like 66.7% (2/3) tumors remained unchanged after 2 weeks of AI. Figure 1B illustrates the changes within Luminal B tumors at baseline after 2 weeks of AI, and that the tumors were increasingly more similar to prototypical Luminal A and normal-like tumors at the 2-week time point. In the control group, although 33.3% (4/12) of Luminal B tumors were reclassified into Luminal A, the majority did not change (Fig. 1C). The difference in intrinsic subtypes after 2 weeks (untreated) was likely due to cases that had close similarity with more than one subtype. In particular, baseline Luminal B tumors that were reclassified into Luminal A had close proximity to prototypical Luminal A tumors as illustrated in Fig. 1D, in contrast to the clear shift from Luminal B to Luminal A seen in the treatment arm. In addition, in the treated samples all PAM50 intrinsic subtypes scores, defined as the correlation coefficient scores to each prototypical intrinsic subtype average gene expression profile (i.e., centroid) changed significantly after 2 weeks of AI, while there were no significant changes in the controls (Supplementary Fig. S2A).

Similar to the POETIC trial, in the NeoAI study, most Luminal B tumors (76.3%, 29/38) were redesignated as Luminal A or normal-like; only 13.2% remained unchanged, while 10.5% were classified as basal-like or Her2-E. Fifty percent (5/10) of Her2-E tumors remained as Her2-E while 20.0% (2/10) were redesignated to Luminal A and 30.0% (3/10) to normal-like (Fig. 1E and F). In this cohort, the changes of the correlation coefficient between baseline and surgery in all intrinsic subtypes were also significant except to basal-like, probably due to the low number of samples in that subtype (Supplementary Fig. S2B).

To further investigate the impact of AI duration on intrinsic subtypes, we tested the correlation of duration of AI with changes of the intrinsic subtype, and there was no statistically significant observed relationship (*P* = 0.19; Supplementary Fig. S3). Overall, the differences in intrinsic subtype classifications were comparable after neoadjuvant endocrine therapy regardless of the duration of treatment, although the total numerical changes in the NeoAI study appeared higher compared with the treated samples in POETIC (67.5% vs. 38.0%), likely due to a higher proportion of Luminal B tumors at baseline in the NeoAI study.

### Changes of gene expression profiles by short- and long-term neoadjuvant AI treatment

**Figure 2.**
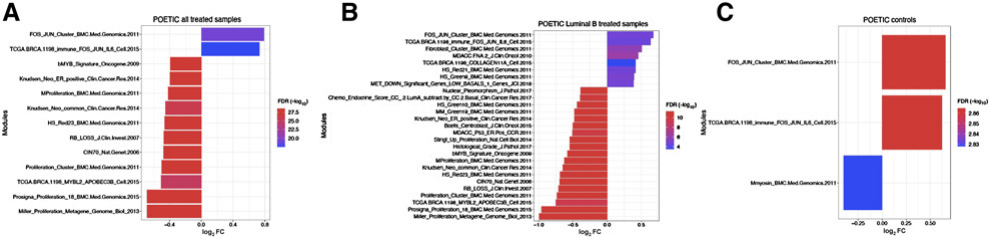
Module scores expression changes in the POETIC cohort. **A**, Barplots showing the significant module scores expression changes between baseline and after 2 weeks of AI in the POETIC dataset for all samples, for Luminal B samples only (**B**) and for controls (**C**). The *x*-axis shows the log_2_FC and the *y*-axis shows the significant module scores that changed. Bars are colored by the degree of significance of the *P* value by paired *t* test. FDR; false discovery rate; log_2_ FC, log_2_ fold change.

Gene expression data in the POETIC subset were computed in module scores according to annotated pathways, immune-response, and selected drug-target response signatures. Two module scores (FOS-JUN modules) were significantly upregulated and 11 significantly downregulated after short-term AI therapy, and these modules included protumorigenic signaling modules associated with proliferation, RB loss, and chromosome instability (Fig. 2A). Within Luminal A samples, 12 also showed a significant change including the upregulation FOS and JUN. Within Luminal B tumors, eight modules' scores increased, and 19 decreased significantly posttreatment (Fig. 2B). As expected for a highly proliferative ER-dependent intrinsic subtype, Luminal B tumors showed a remarkable downregulation of module scores involving proliferation, RB-loss, p53 status, B-cell pathways, and the chemo-endocrine score (CES). Significant upregulation of FOS and JUN module scores was also observed in this subset.

As expected, there were only three module scores significantly different between baseline and surgery in POETIC controls, including the upregulation of FOS and JUN modules (Fig. 2C).

**Figure 3.**
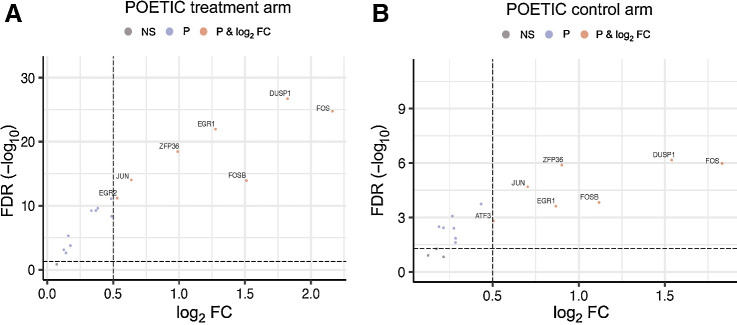
Differential single-gene expression changes between baseline and surgery of the 17 genes included in the FOS and JUN module scores in the POETIC cohort. Differential expression in the treated samples from POETIC subset (**A**) and in the controls (**B**). In red, there are the significant genes by *P* values by paired *t* tests and log_2_FC. FDR, false discovery rate; log_2_ FC, log_2_ fold change; NS, nonsignificant; P, significant by *P* value paired *t* tests; P&log_2_FC, significant by *P* value and log_2_ fold change.

Our group had previously identified the upregulation of *FOS* and *JUN* expression in both treated and control samples as an artefactual effect resulted from preanalytic sample processing due to handling methodology (25, 26). In this study, the expression of the 17 genes from FOS and JUN module scores was explored. The expression of all those genes was strongly correlated at surgery in both POETIC-treated and control samples. Six genes—*JUN*, *FOS*, *FOSB*, *EGR1*, *ZFP36*, and *DUSP1*—showed significantly higher expression in surgical samples in relation to the paired baseline samples in both treated (*P* value all genes < 0.0001, log_2_ FC = 0.5–1.8) and controls (*P* value all genes < 0.0001, log_2_ FC = 0.5–2.2; Fig. 3).

Looking at the changes of expression profiles at a single-gene level from baseline to surgery in the two studies (Supplementary Table S2), a higher number of genes involving proliferation, keratin expression, and endocrine-related pathways like *PGR*, were downregulated in NeoAI when compared with POETIC. More genes from key pathways in breast cancer such as *MAPK* and *PI3K-AKT* (i.e., *IGF1*, *NR4A1* and *NGFR*) or mTOR (*BTG2*; ref. 15) were upregulated in the NeoAI study (Supplementary Table S3).

**Figure 4.**
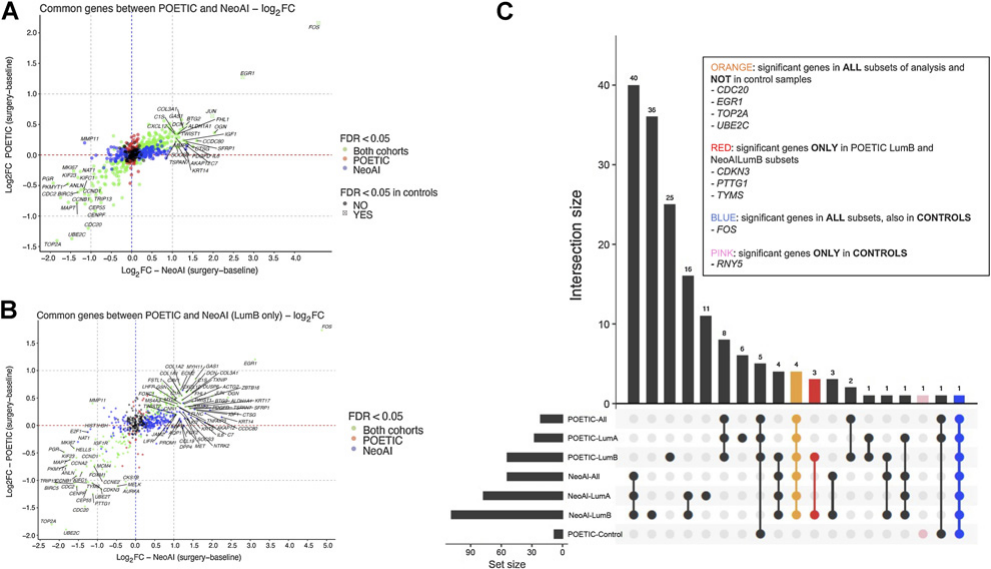
Single-gene expression changes of genes in common between baseline and surgery in the different cohorts. **A,** Scatterplot of differentially expressed genes between baseline and surgery measured by log_2_ FC among the entire short-term and long-term AI cohorts. **B,** Scatterplot of differentially expressed genes between baseline and surgery measured by log_2_ FC among Luminal B–treated tumors only in the short-term and long-term AI cohorts **C,** Boxplot showing the intersections of common genes differentially expressed between baseline and surgery among different combinations of subgroups of sample patients within the POETIC and NeoAI cohorts: all treated patients in POETIC, only treated with Luminal A in POETIC, only treated with Luminal B in POETIC, all patients in NeoAI, only Luminal A in NeoAI, only Luminal B patients in NeoAI, only controls in POETIC. FDR, false discovery rate; log_2_ FC, log_2_ fold change.

The differential expression of genes in common between both datasets are shown in Fig. 4A and B. Genes from FOS-JUN modules *(FOS*, *JUN*, and *ERG1*), *MAPK/ERK*, *PI3K-AKT* and *JAK/STAT* pathways, and *IGF1* involved in tumor growth and resistance to AI, were upregulated in both studies. Consistent to the mechanisms of endocrine therapy, most of the downregulated genes in common were involved in cell-cycle regulation and proliferation. A higher number of genes changed significantly from baseline to surgery after longer-term treatment compared with shorter AI therapy in the overall populations (Fig. 4A) and in Luminal B tumors only (Fig. 4B). In addition, although fold changes were highly correlated between the two datasets, the magnitude of changes for individual genes in POETIC microarray data matrix was smaller compared with the NeoAI Nanostring data matrix.

To investigate gene expression changes under AI treatment relating to the biology of intrinsic subtyping and artefact effect, the list of genes were compared among the following subgroups: (i) All POETIC-treated, (ii) POETIC Luminal A treated, (iii) POETIC Luminal B treated, (iv) POETIC controls, (v) all NeoAI, (vi) NeoAI Luminal A, and (vii) NeoAI Luminal B. Figure 4C shows the exclusive genes that were found significantly different expressed between baseline and surgery in each particular subgroup and the genes in common with the other subgroups, namely intersections. The four common genes that changed significantly in all categories of patients treated with 2 weeks of AI or longer-term AI were *CD20*, *EGR1*, *TOP2A*, and *UBE2C*, all being involved in cell-cycle regulation and proliferation. Noteworthy, only *FOS* was common for all patients including treated and controls.

In a separate analysis, gene expression levels in non-Luminal tumors of NeoAI study were also significantly affected by AI treatment despite being thought to be associated with nonresponse to endocrine therapy. Those changes include the upregulation of *FOS* and *JUN* and the downregulation of some proliferation and endocrine-related genes including *BIRC5*, *MKI67*, and *PGR*.

Finally, to understand the impact of duration of AI on gene expression, multiple *t* tests comparing the changes in gene expression between patients receiving shorter (1–2 months) versus longer (>2 months) AI treatment in the NeoAI dataset and Kruskall–Wallis tests to compare patients grouped in 1–2 months versus >2–6 months versus >6 months, respectively. There were not significant differential changes in gene expression among those categories. We also investigated whether there were positive correlations between the length of AI treatment with the changes in the expression level of those genes associated with an artefactual effect in POETIC control samples. We explored the expression of the significant genes within FOS and JUN modules, namely *FOS*, *JUN*, and *EGR1*, in the NeoAI study. No correlation of gene expression changes (log_2_ FC) with length of AI treatment was observed (*P* value range = 0.68–0.90; Supplementary Fig. S4).

### Impact of longer neoadjuvant endocrine therapy on gene module scores associated with early aromatase resistance between baseline and surgery

Next, we explored whether there was an association between changes of intrinsic subtypes (i.e., from high-risk subtype to lower-risk) with classes of Ki67-level changes (H-H/H-L). All intrinsic subtypes with the capacity of lowering the risk (all except LumA and normal) were classified into “changes” if they turned into a lower-risk intrinsic subtype or “not changes” if they remained the same subtype or turned into a higher-risk subtype. There was a statistically significant association between “no-changes or changes to a higher-risk intrinsic subtype” with H-H Ki67 response category in both subsets (POETIC-treated cohort: 100% of no-changes were classified as H-H tumors and 48.5% of changes being H-H and 51.5% being H-L; *P* = 0.0013; NeoAI study: 58.8% of no-changes were in the H-H group and 41.2% in H-L and 100% of changes in H-L; Fisher exact test *P* < 0.0001).

Most treated Luminal B tumors in the POETIC subset (17/27; 63%), and all Luminal B tumors in the NeoAI study (33/33; 100%) that were reclassified as Luminal A or normal-like changed from high Ki67 at baseline to low Ki67 at surgery (H-L). These data support that these reclassified Luminal B tumors were AI sensitive (Fisher exact test *P* < 0.0001).

**Figure 5.**
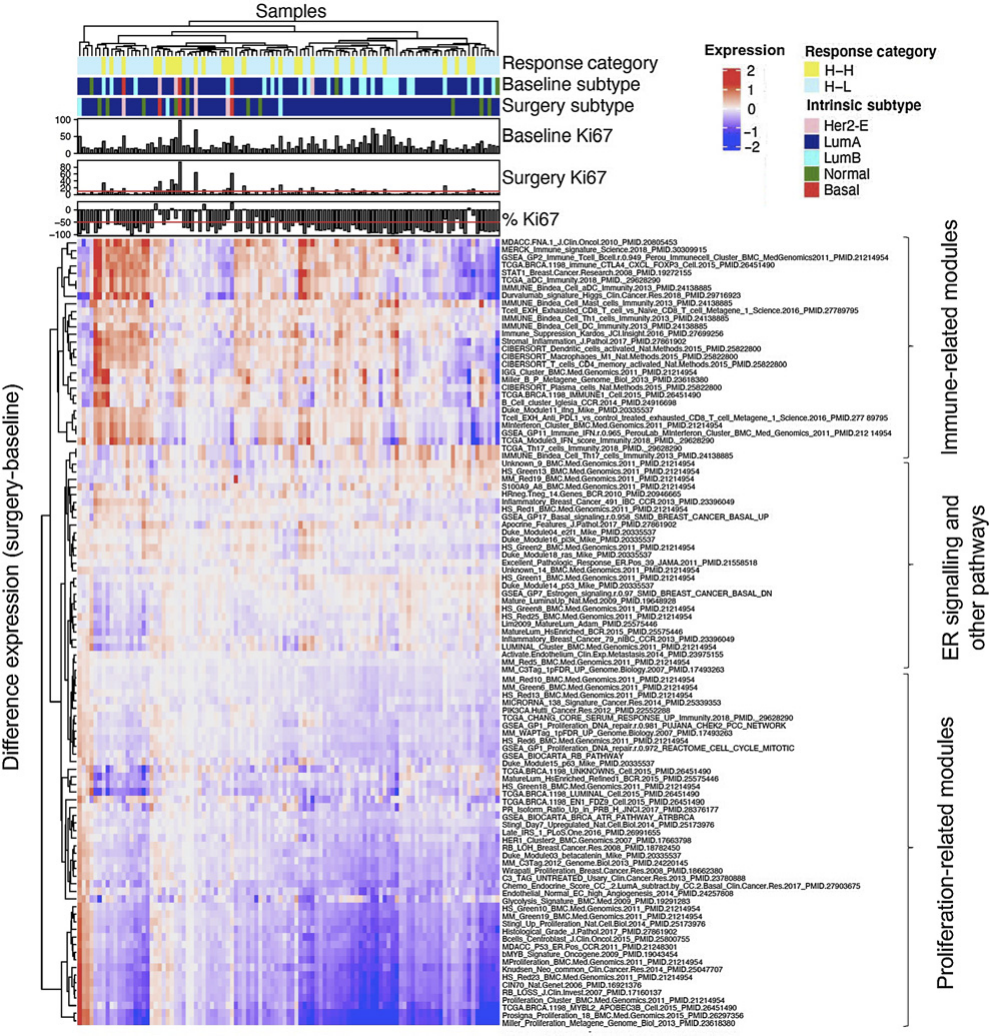
Unsupervised hierarchical clustering showing the difference on gene expression modules scores from baseline to surgery in the POETIC-treated subset (gene expression changes: surgery-baseline). The module scores shown in this heatmap are those selected at baseline by two unpaired SAM analysis between Ki67 H-H versus H–L, categories in the POETIC subset and annotated by the main categories. ER, estrogen receptor; expression, gene expression; H-H, Ki67 High_baseline_- Ki67 High_surgery_; H-L, Ki67 High_baseline_- Ki67 Low_surgery_; Her2-E, Her2 enriched; LumB, Luminal B; LumA, Luminal A; 2 wk; 2-week time point.

Using SAM analysis, we selected 103 candidate gene modules at baseline that were associated with response to early neoadjuvant AI among 105 ER^+^ HER2-negative tumors in the POETIC-treated group. As expected, baseline Ki67 was remarkably higher within Luminal B intrinsic subtype samples with a trend on retaining high Ki67 after 2 weeks of AI compared with Luminal A tumors (Supplementary Fig. S5A). There were 24 immune-related gene modules covering immune-cell pathways, immune-checkpoint component, and IFNγ biology high-expressed at baseline that were associated with early AI resistance (Supplementary Fig. S5A). These gene modules include some genes that have been previously associated with Luminal B–resistant tumors such as *IFNG*, *STAT1*, *IDO1*, *LAG 3*, and *CTLA4* (19). Visualizing the gene expression changes in paired baseline-surgery samples following short AI treatment, there was a general trend observed for downregulation of proliferation-related module scores but otherwise, no changes on expression of the selected modules, including the 24 immune-related gene modules, were associated with differential response to AI (Fig. 5). To assess the differential gene expression changes between responder and nonresponder tumors (H-H vs. H-L), SAM analysis based on changes of the module scores from baseline to surgery was performed in the POETIC subset. Changes in five module scores covering ER signaling, proliferation, and cell cycle were significant (Supplementary Fig. S5B). Supplementary Figure S6 demonstrates the overview of differential changes in gene expression levels (i.e., expression level at surgery minus expression level at baseline) selected by SAM on Ki67 response categories (H-H vs. H-L, *n* = 74) within the NeoAI dataset. Ninety-nine differentially expressed gene changes were selected by SAM (FDR < 0.001; Supplementary Fig. S6). Gene Ontology enrichment analysis showed that genes related to proliferative and cell-cycle pathways were upregulated in the H-H group compared with H-L.

To investigate further the changes of immune-related features associated with resistance to AI by duration of neoadjuvant endocrine therapy, two immune-related module scores were calculated: (i) The “durvalumab signature” (median of *PD-L1*, *LAG3*, *CXCL9*), previously reported to predict response to immunotherapy in melanoma (20) and (ii) the “immune-tolerance signature” (median of *PD-L1*, *LAG3*, *IDO1*), a module score reported by *M.Ellis* group as associated with resistance to AI in Luminal B tumors in the neoadjuvant setting (19).

**Figure 6.**
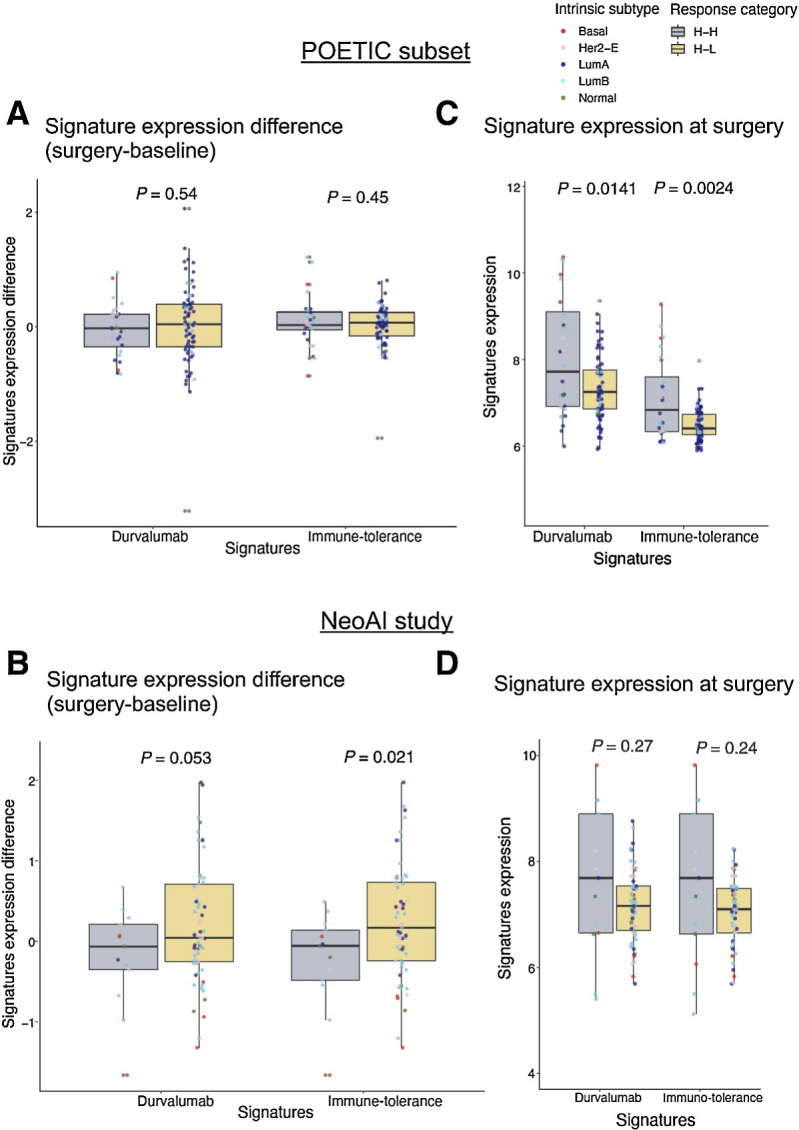
Boxplots showing changes in gene expression from baseline to surgery of the two immune-related signatures (“durvalumab” and “immune-tolerance”) among H-H and H-L Ki67 response categories in the POETIC-treated subset (**A**) and in the NeoAI study (**B**). Boxplots showing gene signature expression of the two immune-related signatures at surgery stratified by H-H and H-L tumors in the POETIC-treated subset (**C**) and in the NeoAI study (**D**). H-H, Ki67 High_baseline_- Ki67 High_surgery_; H-L, Ki67 High_baseline_- Ki67 Low_surgery_; Her2-E, Her2 enriched; LumB, Luminal B; LumA, Luminal A; 2 wk, 2-week time point.

In the POETIC subset, the higher expression of “durvalumab signature” and “immune-tolerance signature” was associated with H-H tumors (Supplementary Fig. S7A). In that setting no significant changes of the signatures' expression from baseline to surgery were seen in either H-H or H-L categories (Fig. 6A; Supplementary Fig. S7A). On the other hand, in the NeoAI cohort, there was also a higher expression of the immune-related signatures in H-H tumors compared with H-L at baseline (Supplementary Fig. S7B) with a differential increase in the expression of “durvalumab signature” (*P* = 0.053) and “Immune-tolerance signature” (*P* = 0.022) from baseline to surgery in H-L tumors compared with H-H tumors (Fig. 6B). Although the expression of both immune signatures at surgery remained significantly higher in H-H tumors compared with H-L in the POETIC subset (Fig. 6C), it was not significantly different after long-term AI therapy (Fig. 6D). The association of the changes of the individual genes included in the two immune-related module scores with resistance to AI, was also assessed. Our results suggest that the enrichment on *PDL1* after longer AI might be the key driving the differences between responders and nonresponders in the NeoAI study (Supplementary Fig. S7C).

Finally, to explore the impact of AI duration on the expression of these two early endocrine-resistance immune-module scores, we looked at the correlation of their expression with time under AI in the NeoAI study. There was no correlation between the changes on their expression from baseline to surgery (log_2_FC) with duration of AI (Supplementary Fig. S8).

### Impact of the significant molecular changes under neoadjuvant aromatase inhibitor treatment on survival

To assess the clinical impact of the observed findings, we tested the association of the significant features described previously with patient survival data, in each of the two datasets as follows: (i) changes in the correlation coefficient scores to prototypical intrinsic subtype centroids from baseline to surgery, (ii) significant changes that associated with resistance to AI, (iii) significant changes from baseline to surgery in all tumors, (iv) significant changes from baseline to surgery in Lumina B tumors.

Results are shown in Supplementary Table S4 (POETIC) and Supplementary Table S5 (NeoAI). First, the increase of correlation score to Luminal B centroid was associated with worse survival in both datasets, while the increase of the correlation scores to Luminal A and normal centroids were associated with better survival. These findings are in line with the observed association of changes in intrinsic subtype with response to AI. Meanwhile, most of the changes that were associated with resistance to AI (H-H tumors) and a subset of the reported significant changes from baseline to surgery found in both datasets, were associated with differential survival. There were no statistically significant associations of the immune features such as the increase of *LAG3*, and the increase of “durvalumab” signature expression with differential outcome.

Our results suggest that some of the molecular changes that were associated with resistance to AI, particularly those associated with significant patient survival may be evaluated further as predictive and prognostic biomarkers.

## Discussion

Although AI treatment is the standard of care and most effective therapy for postmenopausal women with early ER^+^ breast cancer, recurrence to AIs is still a main issue. Molecular characterization of gene expression profiles that occur in response to neoadjuvant AIs is necessary to identify mechanisms of resistance. This study was designed to understand the complexities of RNA-based expression changes under short time exposure to ET and to compare them with those that occur under longer-term AI therapy. The main observations from this study are: (i) most AI-sensitive Luminal B and Her2-E tumors change their intrinsic subtype within just 2 weeks of treatment, mainly from Luminal B toward Luminal A or normal-like; these changes are associated with differential response to AI and outcome; (ii) in contrast, longer AI treatment may induce additional and greater gene expression changes than 2 weeks only; (iii) confirmation that FOS- and JUN-related gene modules and single-gene expression upregulation might be explained by sampling manipulation and not just by AI treatment; (iv) breast cancer tumors showing early resistance to AI are characterized by a greater expression of immune-checkpoint component, immune-cell enrichment and proliferation, and these signatures were more impacted by longer AI treatment.

ER^+^/HER2^−^ tumors should not be considered and treated as a homogeneous disease; thus, the analysis of intrinsic subtypes may help to predict response to therapy even in early-stage breast cancer (27–29). Previous data have shown that exposure to ET might lead to profound changes on intrinsic subtypes, mainly from Luminal B or Her2-E to Luminal A (30). However, most of those studies used long-term treatment and included a low proportion of “high-risk” tumors—the majority were Luminal A at baseline. From a biological perspective, our study also shows that most Luminal B or Her2-E tumors with the potential of lowering their proliferative biology will change their intrinsic subtype to Luminal A or normal-like within just 2 weeks of treatment, but more “endocrine-resistant” breast cancer such as basal-like and some Luminal B and Her2-E will not change despite prolonged AI treatment. On the basis of prior studies, Luminal A and Luminal B baseline tumors are more likely to respond to endocrine therapy than other intrinsic subtypes (30, 31); however, our study also suggests that changes toward a lower-risk subtype correlate with sensitivity to AI treatment and better survival, beyond baseline intrinsic subtypes. Thus, an early reassessment of the intrinsic subtype at the 2-week time point could be essential to distinguish those “sensitive” tumors from the “resistant” to optimize clinical management following surgery.

Clustering gene expression into signatures/modules catches the biology of main cancer pathways and can be more easily associated with clinical outcome (29, 32–35). In our study, gene expression changes were far more discrete after short-term AI compared with longer AI treatment. As expected, most of the downregulated module scores in the POETIC subset involved a decrease of the “high-risk” characteristics toward a “lower-risk” profile. The general transition to a "lower proliferative" phenotype seen in the POETIC cohort with slight changes on the rest of the genes might be explained by the dominant impact of AI on proliferation and cell-cycle pathways, also in agreement with the rapid changes observed in intrinsic subtypes. For example, RB protein—a critical protein in cell-cycle regulation, prevents unscheduled entry into the mitotic cell cycle. RB-loss would impede the antiproliferative effect of AI treatment and consequently, the downregulation seen under AI would revert this negative feedback (36, 37). Second, a PAM50-based CES in ER^+^/HER2^−^ early disease is capable of predicting response to ET in comparison with chemotherapy (38). Higher CES values are associated with endocrine sensitivity and chemoresistance, hence, the estrogen deprivation occurring under AI treatment would lead to a drop on this score.

The common differential genes observed for both short- and long-term AI-treated cohorts were also involved mainly in cell-cycle, dedifferentiation, and proliferation pathways, reflecting the main molecular features that would be affected by hormone deprivation regardless duration of treatment. In agreement to previous studies, the effect of longer AI treatment was also seen as a general but deeper downregulation of proliferation and endocrine-related genes (15, 20, 24, 35). Noteworthy, only after longer-term neoadjuvant AI, some genes involved in key signaling pathways associated with AI resistance, such as *MAPK* and *PI3K/AKT/mTOR*, showed increased expression, and thus a possible mechanism of ER activation in a ligand-independent manner (39–42). Prior studies have also suggested that ET could have an immune effect leading to an enrichment of tumor-infiltrating cells and immune-related characteristics (43–45) as well as the importance of tumor microenvironment in cancer progression and therapeutic responses (46). Here, some stromal-related module scores and single genes within Luminal B samples increased their expression significantly at surgery in both datasets while only long-term AI had an immune-enrichment effect with a significant upregulation of genes involving inflammatory chemokines or immune pathways such as SOCS3, JAK/STAT signaling and other chemokines and ILs, as well as the two immune-related gene modules in H-L tumors. In this article, we have also reported the prognostic value of some of those gene expression changes induced by 2 weeks and longer AI treatment, respectively, suggesting that the clinical utility of these molecular changes as prognostic or predictive biomarkers to treatment should be studied further. The sample size was small and thus a bigger study is warranted.

Furthermore, our group had previously characterized for the first time, the increase of expression of some genes, such as *FOS* and *JUN* as an artefactual effect resulted from preanalytic sample processing (24). In the current study, we demonstrate that FOS- and JUN-related module scores increase significantly from baseline to surgery in both NeoAI and POETIC-treated cohorts, as well as in surgical samples from POETIC nontreated patients. This confirms that the upregulation of the expression of several genes included in those module scores is induced by sample manipulation rather than only by AI treatment. In the absence of a control group, these artefactual changes would likely be considered as an exclusive effect of AI what might be relevant to all archival collections of ER^+^ breast cancer.

Finally, the POETIC trial has previously validated Ki67 as a prognostic marker showing that patients whose Ki67 remains “HIGH” (≥ 10%) after 2 weeks of AI treatment have substantially poorer prognosis than those with a “HIGH” baseline Ki67 which is markedly reduced to “LOW” (<10%; refs. 15, 20). Thus, differential gene expression between H-H and H-L response groups is essential to distinguish those patients who might benefit the most from AI treatment from those who would not. Most of the H-H tumors in our POETIC cohort were Luminal B at baseline and in the NeoAI being Luminal B, basal-like, and Her2-E. As expected, the upregulation of cell-cycle and proliferation-related genes and modules from baseline to surgery was associated with resistance to AI as measured by changes in Ki67 value and worst patient survival outcome.

Luminal tumors are usually known to be less immunogenic than Her2-E and basal-like subtypes (46), but those with higher immunogenicity have been correlated with poor prognosis or response to ET therapy (3, 19). Anurag and colleagues have recently demonstrated that immune checkpoint–related genes are upregulated in most Luminal B tumors that show poor response to ET as measured by higher Ki67 (19). Another study has shown association of AI treatment with a variety of autoimmune disorders in some patients, suggesting a clear effect on immune cells and tumor immunity of AI therapy (47). However, the magnitude of that effect is still unknown and a clinical study comparing changes on immune-related features after different length of AI to understand the real impact of AI treatment could be important for clinical management. In our study, we looked at both baseline characteristics and changes on immune-related features under different lengths of AI and observed an association of high expression of immune-related module scores measured at 2 weeks of AI with nonresponder tumors in POETIC but not at the surgical timepoint after longer term AI in the NeoAI, probably due to the significant upregulation observed on the expression of those signatures after longer treatment. There was no statistically significant association of the increase in some of those immune-related features with survival in the NeoAI cohort, but a larger study would be needed to properly refute the hypothesis. Taking together our results and those from the literature, a small subgroup of ER^+^/HER2^−^ breast cancer could potentially benefit from immunotherapy, currently approved for metastatic triple-negative breast cancer and having been tested in other subsets (48–50). Although the assessment of immune characteristics at baseline could be informative to detect mechanism of resistance to AI, further investigation is still necessary to understand the utility of the analysis of immune-related module scores in surgical samples of patients treated with long-term AI and whether the enrichment of some immune-related signatures in H-L tumors after longer AI treatment has a role in acquired therapy resistance and survival.

Our study has some limitations and strengths. First, we analyzed data from two subsets with very different backgrounds, data collection and analytic methodology. However, our targeted analyses were focused on pathways/modules, facilitating the evaluation of changes under AI treatment and comparison among datasets. Moreover, we included a control group that enabled the distinction between real impact of AI therapy and artefactual effect derived from sample manipulation. Second, although we aimed to compare short- versus long-term AI treatment, the NeoAI dataset includes patients treated with a huge range of AI therapy duration in a presurgical setting. Additional whole transcriptome work in a much larger subset of the POETIC treatment arm is ongoing to better understand the diversity of intrinsic resistance mechanisms to AI treatment and to increase the power of our survival analyses. This work will also include genomic analysis to determine if there is subset of resistant Luminal patients with immune tolerance and high antigenicity that could benefit from immunotherapy. However, this is a modest but real-world cohort and has a unique value to assess global gene expression data from both pre- and post-AI treatment as defined in the clinical practice. Last but not least, this is the first study to our knowledge to investigate and compare the molecular changes from short- and long-term AI treatment.

## Conclusion

Short- and longer-term AI treatment have similar effects to the changes of the intrinsic subtype's classifications. However, longer neoadjuvant AI treatment leads to deeper impact on molecular characteristics (changes in gene expression) beyond intrinsic subtypes, including signatures covering immune-checkpoint component reported previously associated with AI early resistant tumors. Some of the observed changes, such as changes in the intrinsic subtypes or enrichment of immune features, were shown not only associated with response to AI but also with patient outcome, thus providing a supporting rationale to consider the continuation with ET for higher-risk tumors if changes in transcriptional gene expression signatures are desired. Finally, further investigation on the use of immune-checkpoint component inhibition in this setting is warranted.

## Supplementary Material

Supplementary Figure

Supplementary Data

## References

[1] Selli C , Turnbull AK, Pearce DA, Li A, Fernando A, Wills J, . Molecular changes during extended neoadjuvant letrozole treatment of breast cancer: distinguishing acquired resistance from dormant tumours. Breast Cancer Res 2019;21:2. 30616553 10.1186/s13058-018-1089-5 PMC6323855

[2] Miller TW , Balko JM, Fox EM, Ghazoui Z, Dunbier A, Dowsett M, . ERα-dependent E2F transcription can mediate resistance to estrogen deprivation in human breast cancer. Cancer Discov 2011;1:338–51. 22049316 10.1158/2159-8290.CD-11-0101 PMC3204388

[3] Dunbier AK , Ghazoui Z, Anderson H, Salter J, Nerurkar A, Osin P, . Molecular profiling of aromatase inhibitor – treated postmenopausal breast tumors identifies immune-related correlates of resistance. Clin Cancer Res 2013;19:2775–86. 23493347 10.1158/1078-0432.CCR-12-1000

[4] Dowsett M , Ellis MJ, Dixon JM, Gluz O, Robertson J, Kates R, . Evidence-based guidelines for managing patients with primary ER+ HER2− breast cancer deferred from surgery due to the COVID-19 pandemic. NPJ Breast Cancer 2020;6:21. 32550266 10.1038/s41523-020-0168-9 PMC7280290

[5] Sørlie T , Tibshirani R, Parker J, Hastie T, Marron JS, Nobel A, . Repeated observation of breast tumor subtypes in independent gene expression data sets. Proc Natl Acad Sci U S A 2003;100:8418–23. 12829800 10.1073/pnas.0932692100 PMC166244

[6] Curtis C , Shah SP, Chin SF, Turashvili G, Rueda OM, Dunning MJ, . The genomic and transcriptomic architecture of 2,000 breast tumours reveals novel subgroups. Nature 2012;486:346–52. 22522925 10.1038/nature10983 PMC3440846

[7] Parker JS , Mullins M, Cheang MC, Leung S, Voduc D, Vickery T, . Supervised risk predictor of breast cancer based on intrinsic subtypes. J Clin Oncol 2009;27:1160–7. 19204204 10.1200/JCO.2008.18.1370 PMC2667820

[8] Bernard PS , Parker JS, Mullins M, Cheang MCU, Leung S, Voduc D, . Supervised risk predictor of breast cancer based on intrinsic subtypes. J Clin Oncol 2009;27:1160–7. 19204204 10.1200/JCO.2008.18.1370 PMC2667820

[9] Martín M , Prat A, Rodríguez-Lescure Á, Caballero R, Ebbert MTW, Munárriz B, . PAM50 proliferation score as a predictor of weekly paclitaxel benefit in breast cancer. Breast Cancer Res Treat 2013;138:457–66. 23423445 10.1007/s10549-013-2416-2 PMC3608881

[10] Poudel P , Nyamundanda G, Patil Y, Cheang MCU, Sadanandam A. Heterocellular gene signatures reveal luminal-A breast cancer heterogeneity and differential therapeutic responses. NPJ Breast Cancer 2019;5:21. 31396557 10.1038/s41523-019-0116-8 PMC6677833

[11] Ellis MJ , Suman VJ, Hoog J, Lin L, Snider J, Prat A, . Randomized phase II neoadjuvant comparison between letrozole, anastrozole, and exemestane for postmenopausal women with estrogen receptor-rich stage 2 to 3 breast cancer: clinical and biomarker outcomes and predictive value of the baseline PAM50-based intrinsic subtype - ACOSOG Z1031. J Clin Oncol 2011;29:2342–9. 21555689 10.1200/JCO.2010.31.6950 PMC3107749

[12] Dowsett M , Smith IE, Ebbs SR, Dixon JM, Skene A, Griffith C, . Short-term changes in Ki-67 during neoadjuvant treatment of primary breast cancer with anastrozole or tamoxifen alone or combined correlate with recurrence-free survival. Clin Cancer Res 2005;11:951–9. 15701892

[13] Dowsett M , Nielsen TO, A'Hern R, Bartlett J, Coombes RC, Cuzick J, . Assessment of Ki67 in breast cancer: recommendations from the international Ki67 in breast cancer working Group. J Natl Cancer Inst 2011;103:1656–64. 21960707 10.1093/jnci/djr393 PMC3216967

[14] Ellis MJ , Suman VJ, Hoog J, Goncalves R, Sanati S, Creighton CJ, . Ki67 proliferation index as a tool for chemotherapy decisions during and after neoadjuvant aromatase inhibitor treatment of breast cancer: results from the American college of surgeons oncology group Z1031 trial (alliance). J Clin Oncol 2017;35:1061–9. 28045625 10.1200/JCO.2016.69.4406 PMC5455353

[15] Gao Q , López-Knowles E, Cheang MCU, Morden J, Ribas R, Sidhu K, . Impact of aromatase inhibitor treatment on global gene expression and its association with antiproliferative response in ER+ breast cancer in postmenopausal patients. Breast Cancer Res 2019;22:2. 31892336 10.1186/s13058-019-1223-z PMC6938628

[16] Leal MF , Haynes BP, Schuster E, Yeo B, Afentakis M, Zabaglo L, . Early enrichment of ESR1 mutations and the impact on gene expression in presurgical primary breast cancer treated with aromatase inhibitors. Clin Cancer Res 2019;25:7485–96. 31548345 10.1158/1078-0432.CCR-19-1129

[17] Fan C , Prat A, Parker JS, Liu Y, Carey LA, Troester MA, . Building prognostic models for breast cancer patients using clinical variables and hundreds of gene expression signatures. BMC Med Genomics 2011;4:3. 21214954 10.1186/1755-8794-4-3 PMC3025826

[18] Waggott D , Chu K, Yin S, Wouters BG, Liu FF, Boutros PC, . An extensible R package for the pre-processing of nanostring mRNA and miRNA data. Bioinformatics 2012;28:1546–8. 22513995 10.1093/bioinformatics/bts188 PMC3356845

[19] Anurag M , Zhu M, Huang C, Vasaikar S, Wang J, Hoog J, . Immune checkpoint profiles in luminal B breast cancer (Alliance). J Natl Cancer Inst 2019;112:737–46. 10.1093/jnci/djz213 PMC7805027 31665365

[20] Higgs BW , Morehouse CA, Streicher K, Brohawn PZ, Pilataxi F, Gupta A, . Interferon gamma messenger RNA Signature in tumor biopsies predicts outcomes in patients with non–small cell lung carcinoma or urothelial cancer treated with durvalumab. Clin Cancer Res 2018;24:3857–66. 29716923 10.1158/1078-0432.CCR-17-3451

[21] Buus R , Szijgyarto Z, Schuster EF, Xiao H, Haynes BP, Sestak I, . Development and validation for research assessment of Oncotype DX® Breast Recurrence Score, EndoPredict® and Prosigna®. NPJ Breast Cancer 2021;7:15. 33579961 10.1038/s41523-021-00216-w PMC7881187

[22] Smith I , Robertson J, Kilburn L, Wilcox M, Evans A, Holcombe C, . Long-term outcome and prognostic value of Ki67 after perioperative endocrine therapy in postmenopausal women with hormone-sensitive early breast cancer (POETIC): an open-label, multicentre, parallel-group, randomised, phase 3 trial. Lancet Oncol 2020;21:1443–54. 33152284 10.1016/S1470-2045(20)30458-7 PMC7606901

[23] Tusher VG , Tibshirani R, Chu G. Significance analysis of microarrays applied to the ionizing radiation response. Proc Natl Acad Sci U S A 2001;98:5116–21. 11309499 10.1073/pnas.091062498 PMC33173

[24] Gu Z , Eils R, Schlesner M. Complex heatmaps reveal patterns and correlations in multidimensional genomic data. Bioinformatics 2016;32:2847–9. 27207943 10.1093/bioinformatics/btw313

[25] Gao Q , López-Knowles E, Morden J, Ribas R, Sidhu K, U Cheang MC, . Major impact of sampling methodology on gene expression in estrogen receptor–positive breast cancer. JNCI Cancer Spectr 2018;2:pky005. 31360844 10.1093/jncics/pky005 PMC6649758

[26] López-Knowles E , Gao Q, Cheang MCU, Morden J, Parker J, Martin LA, . Heterogeneity in global gene expression profiles between biopsy specimens taken peri-surgically from primary ER-positive breast carcinomas. Breast Cancer Res 2016;18:39. 27036195 10.1186/s13058-016-0696-2 PMC4818440

[27] Cejalvo JM , Pascual T, Fernández-Martínez A, Brasó-Maristany F, Gomis RR, Perou CM, . Clinical implications of the non-luminal intrinsic subtypes in hormone receptor-positive breast cancer. Cancer Treat Rev 2018;67:63–70. 29763779 10.1016/j.ctrv.2018.04.015

[28] Adamo B , Bellet M, Paré L, Pascual T, Vidal M, Pérez Fidalgo JA, . Oral metronomic vinorelbine combined with endocrine therapy in hormone receptor-positive HER2-negative breast cancer: SOLTI-1501 VENTANA window of opportunity trial. Breast Cancer Res 2019;21:108. 31533777 10.1186/s13058-019-1195-z PMC6751874

[29] Bertucci F , Finetti P, Goncalves A, Birnbaum D. The therapeutic response of ER+/HER2− breast cancers differs according to the molecular Basal or Luminal subtype. NPJ Breast Cancer 2020;6:1–7. 32195331 10.1038/s41523-020-0151-5 PMC7060267

[30] Prat A , Cheang MC, Galván P, Nuciforo P, Paré L, Adamo B, . Prognostic value of intrinsic subtypes in hormone receptor-positive metastatic breast cancer treated with letrozole with or without lapatinib. JAMA Oncol 2016;2:1287–94. 27281556 10.1001/jamaoncol.2016.0922

[31] Pascual T , Martin M, Fernández-Martínez A, Paré L, Alba E, Rodríguez-Lescure Á, . A pathology-based combined model to identify PAM50 non-luminal intrinsic disease in hormone receptor-positive HER2-negative breast cancer. Front Oncol 2019;9:303. 31106144 10.3389/fonc.2019.00303 PMC6498671

[32] Yosef N , Shalek AK, Gaublomme JT, Jin H, Lee Y, Awasthi A, . Dynamic regulatory network controlling TH 17 cell differentiation. Nature 2013;496:461–8. 23467089 10.1038/nature11981 PMC3637864

[33] Jojic V , Shay T, Sylvia K, Zuk O, Zuk O, Sun X, . Identification of transcriptional regulators in the mouse immune system. Nat Immunol 2013;14:633–43. 23624555 10.1038/ni.2587 PMC3690947

[34] Paul F , Arkin Y, Giladi A, Jaitin DA, Kenigsberg E, Keren-Shaul H, . Transcriptional heterogeneity and lineage commitment in myeloid progenitors. Cell 2015;163:1663–77. 26627738 10.1016/j.cell.2015.11.013

[35] Saelens W , Cannoodt R, Saeys Y. A comprehensive evaluation of module detection methods for gene expression data. Nat Commun 2018;9:1090. 29545622 10.1038/s41467-018-03424-4 PMC5854612

[36] Witkiewicz AK , Knudsen ES. Retinoblastoma tumor suppressor pathway in breast cancer: prognosis, precision medicine, and therapeutic interventions. Breast Cancer Res 2014;16:207. 25223380 10.1186/bcr3652 PMC4076637

[37] Rani A , Stebbing J, Giamas G, Murphy J. Endocrine resistance in hormone receptor positive breast cancer–from mechanism to therapy. Front Endocrinol 2019;10:245. 10.3389/fendo.2019.00245 PMC6543000 31178825

[38] Prat A , Lluch A, Turnbull AK, Dunbier AK, Calvo L, Albanell J, . A PAM50-based chemoendocrine score for hormone receptor-positive breast cancer with an intermediate risk of relapse. Clin Cancer Res 2017;23:3035–44. 27903675 10.1158/1078-0432.CCR-16-2092 PMC5449267

[39] Gul A , Leyland-Jones B, Dey N, De P. A combination of the PI3K pathway inhibitor plus cell cycle pathway inhibitor to combat endocrine resistance in hormone receptor-positive breast cancer: a genomic algorithm-based treatment approach. Am J Cancer Res 2018;8:2359–76. 30662797 PMC6325472

[40] Vasan N , Toska E, Scaltriti M. Overview of the relevance of PI3K pathway in HR-positive breast cancer. Ann Oncol 2019;30:x3–11. 31859348 10.1093/annonc/mdz281 PMC6923788

[41] Sundaramoorthy S , Devanand P, Ryu MS, Song KY, Noh DY, Lim IK. TIS21/BTG2 inhibits breast cancer growth and progression by differential regulation of mTORc1 and mTORc2–AKT1–NFAT1–PHLPP2 signaling axis. J Cancer Res Clin Oncol 2018;144:1445–62. 29808317 10.1007/s00432-018-2677-6 PMC11813427

[42] Braicu C , Buse M, Busuioc C, Drula R, Gulei D, Raduly L, . A comprehensive review on MAPK: a promising therapeutic target in cancer. Cancers 2019;11:1618. 31652660 10.3390/cancers11101618 PMC6827047

[43] Mello-Grand M , Singh V, Ghimenti C, Scatolini M, Regolo L, Grosso E, . Gene expression profiling and prediction of response to hormonal neoadjuvant treatment with anastrozole in surgically resectable breast cancer. Breast Cancer Res Treat 2010;121:399–411. 20428938 10.1007/s10549-010-0887-y

[44] Louault K , Bonneaud TL, Séveno C, Gomez-Bougie P, Nguyen F, Gautier F, . Interactions between cancer-associated fibroblasts and tumor cells promote MCL-1 dependency in estrogen receptor-positive breast cancers. Oncogene 2019;38:3261–73. 30631150 10.1038/s41388-018-0635-z PMC6756023

[45] Sobral-Leite M , Salomon I, Opdam M, Kruger DT, Beelen KJ, Van Der Noort V, . Cancer-immune interactions in ER-positive breast cancers: PI3K pathway alterations and tumor-infiltrating lymphocytes. Breast Cancer Res 2019;21:90. 31391067 10.1186/s13058-019-1176-2 PMC6686400

[46] Helleman J , Jansen MPHM, Ruigrok-Ritstier K, van Staveren IL, Look MP, Meijer-van Gelder ME, . Association of an extracellular matrix gene cluster with breast cancer prognosis and endocrine therapy response. Clin Cancer Res 2008;14:5555–64. 18765548 10.1158/1078-0432.CCR-08-0555

[47] Zarkavelis G , Kollas A, Kampletsas E, Vasiliou V, Kaltsonoudis E, Drosos A, . Aromatase inhibitors induced autoimmune disorders in patients with breast cancer: a review. J Adv Res 2016;7:719–26. 28275510 10.1016/j.jare.2016.04.001 PMC5328027

[48] Schmid P , Rugo HS, Adams S, Schneeweiss A, Barrios CH, . IMpassion130 Investigators. Atezolizumab plus nab-paclitaxel as first-line treatment for unresectable, locally advanced or metastatic triple-negative breast cancer (IMpassion130): updated efficacy results from a randomised, double-blind, placebo-controlled, phase 3 trial. Lancet Oncol 2020;21:44–59. 31786121 10.1016/S1470-2045(19)30689-8

[49] Tolaney SM , Barroso-Sousa R, Keenan T, Li T, Trippa L, Vaz-Luis I, . Effect of eribulin with or without pembrolizumab on progression-free survival for patients with hormone receptor-positive, ERBB2-negative metastatic breast cancer: a randomized clinical trial. JAMA Oncol 2020;6:1598–605. 32880602 10.1001/jamaoncol.2020.3524 PMC7489368

[50] Emens LA , Esteva FJ, Beresford M, Saura C, De Laurentiis M, Kim SB, . Trastuzumab emtansine plus atezolizumab versus trastuzumab emtansine plus placebo in previously treated, HER2-positive advanced breast cancer (KATE2): a phase 2, multicentre, randomised, double-blind trial. Lancet Oncol 2020;21:1283–95. 33002436 10.1016/S1470-2045(20)30465-4

