# Supplementary material for: Impact of Duration of Neoadjuvant Aromatase Inhibitors on Molecular Expression Profiles in Estrogen Receptor–positive Breast Cancers
Source: Clin Cancer Res. 2022 Mar 14;28(6):1217–28. doi: 10.1158/1078-0432.CCR-21-2718 (PMC7612503; doi:10.1158/1078-0432.CCR-21-2718)

**SUPPLEMENTARY FIGURES**

**Supplementary figure S1**. Overview of the study including POETIC and NeoAI cohorts.


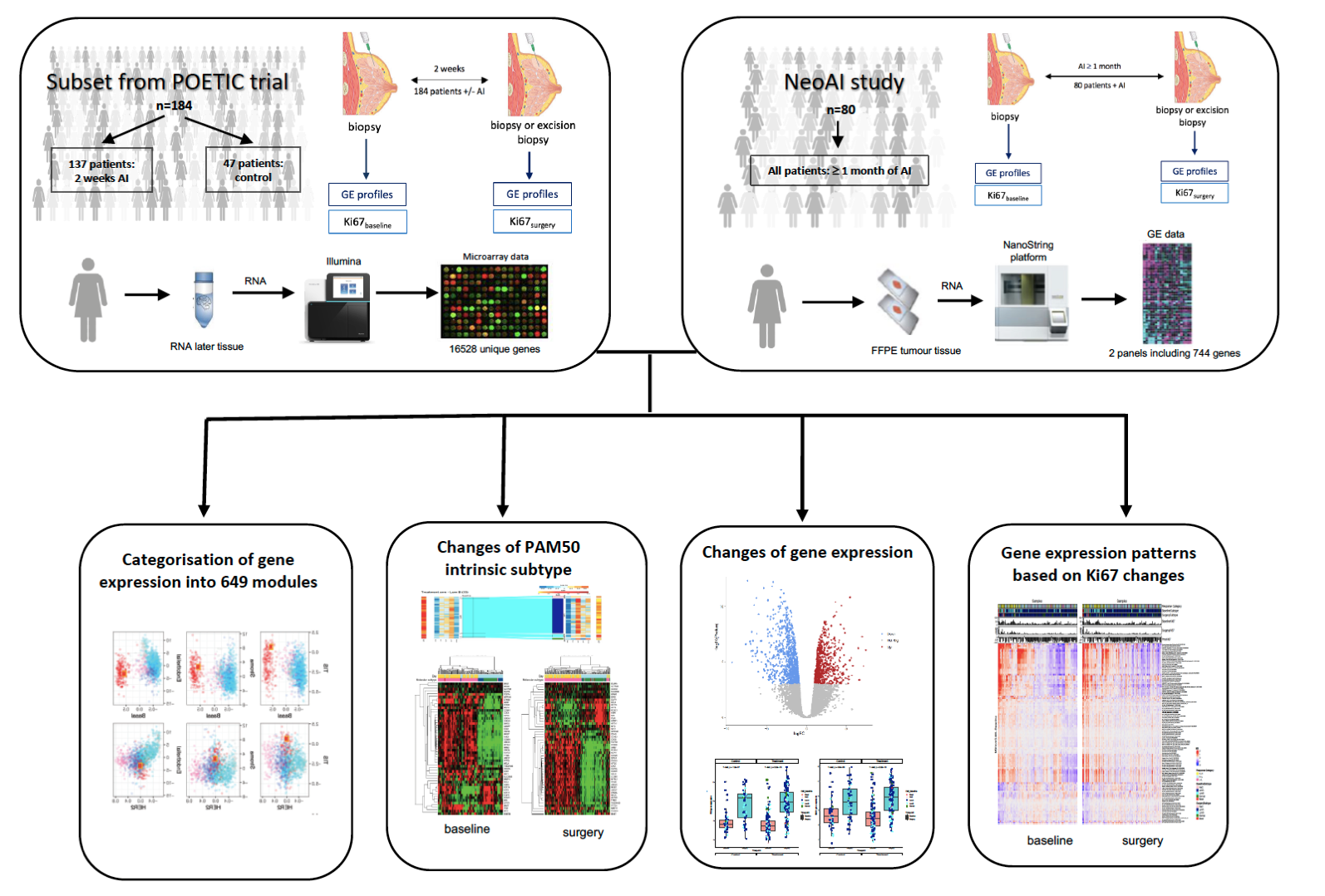


**Abbreviations:** n: number, GE: Gene expression, AI: Aromatase inhibitors, FFPE: Formalin-Fixed Paraffin-Embedded samples

**Supplementary figure S2.** Changes on subtype correlation coefficients **A.** Changes on subtype correlation coefficients in treated and control samples in the POETIC cohort. Red lines: Tumours showing change in intrinsic subtype after 2-weeks (Treated: 38.0%; Controls: 23.4%). **B.** Changes on subtype correlation coefficients in the NeoAI study. Red line: Tumours showing changes in intrinsic subtype after neoadjuvant treatment (NeoAI study: 67.5%). **Abbreviations:** Her2-E: Her2 enriched, LumB: Luminal B, LumA: Luminal A.

**
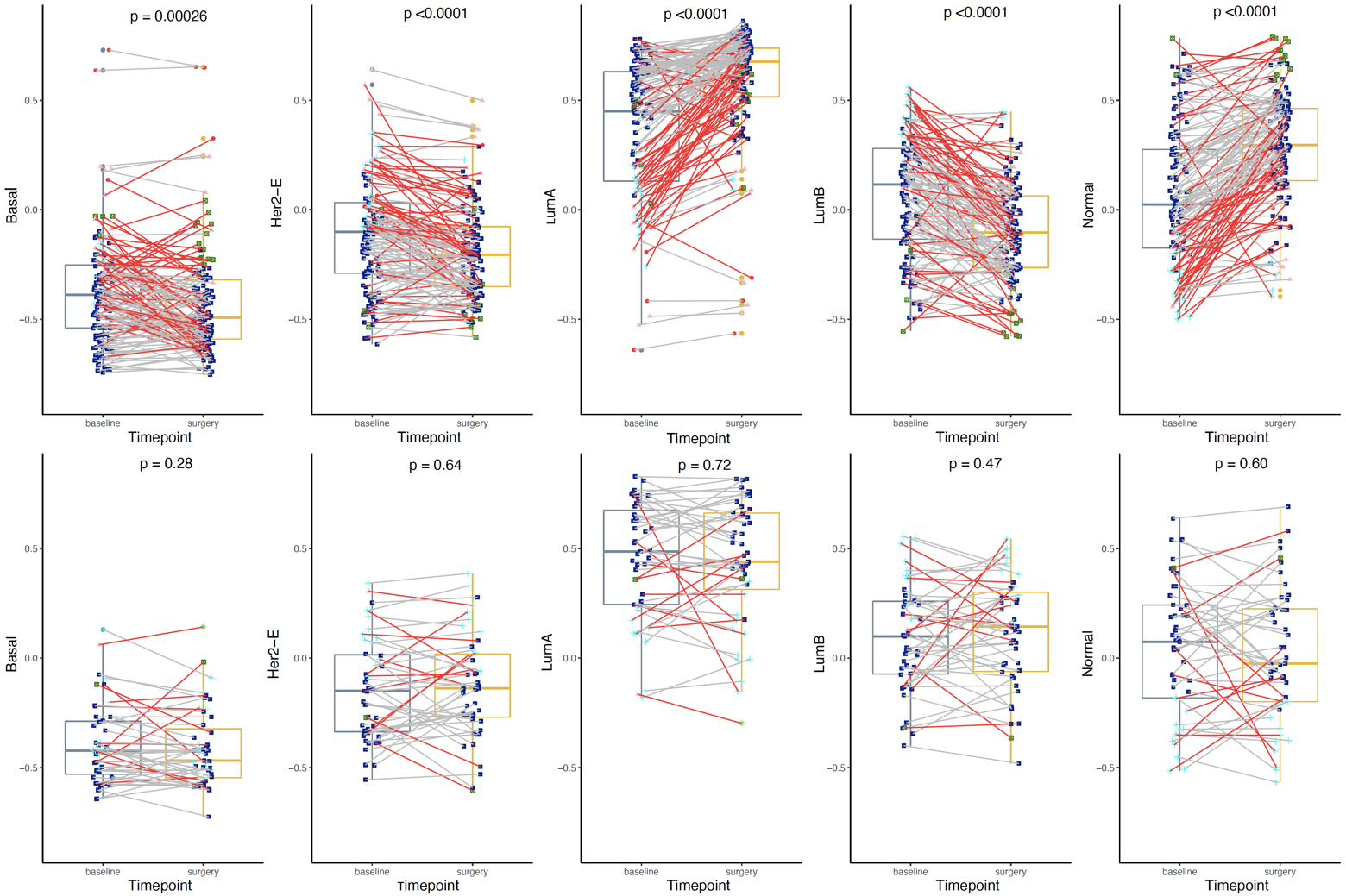
A.**

POETIC treatment arm

POETIC control arm

**
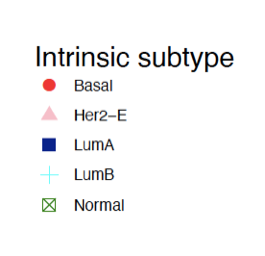
**

**B.**

**
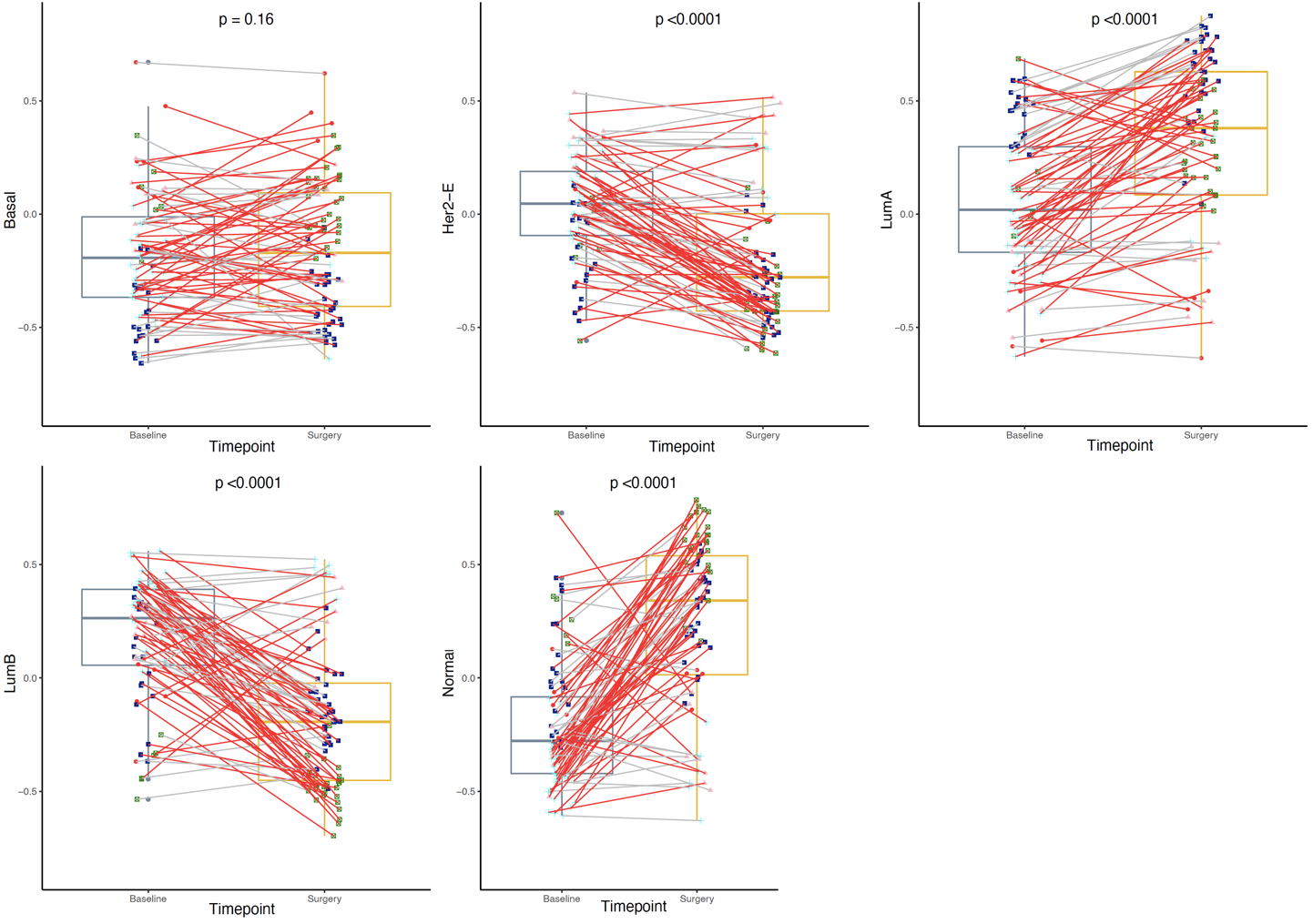
**

NeoAI study

**
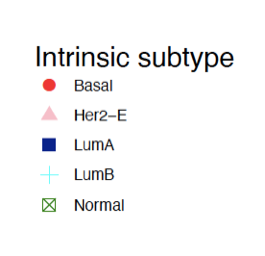
**

**Supplementary figure S3.** Association test of the median number of days under AI treatment and changes on PAM50 intrinsic subtype (yes/no).

**Abbrevietions**: Her2-E: Her2 enriched, LumB: Luminal B, LumA: Luminal A


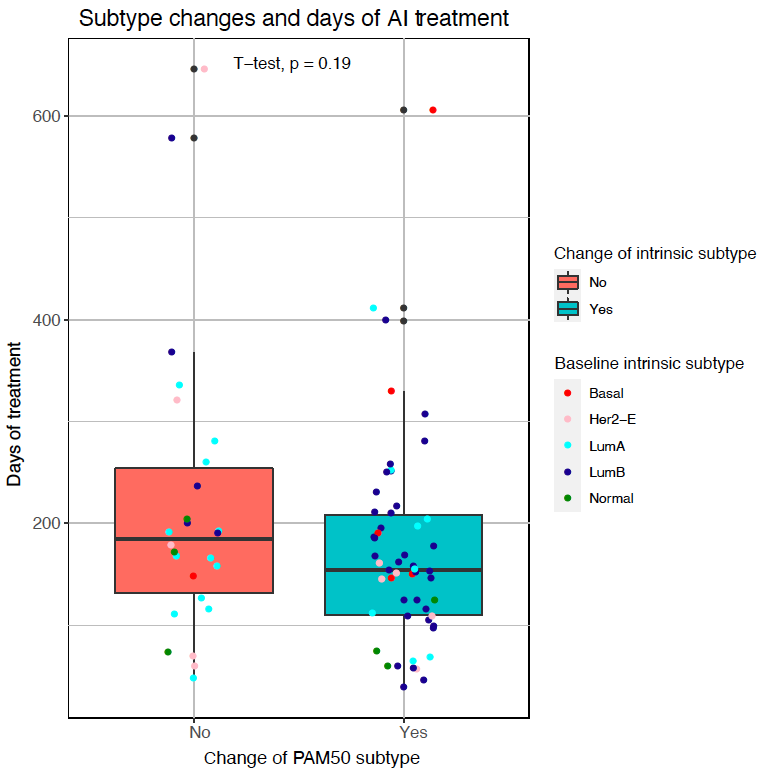


**Supplementary figure S4.** Spearman Rank-Order Correlation test of the changes of expression from baseline to surgery (Log2FC) under AI treatment of available genes from FOS and JUN modules in the POETIC cohort (FOS, JUN and EGR1) with time in the NeoAI study. **Abbreviations**: FC: Fold change, Her2-E: Her2-enriched, LumB: Luminal B, LumA: Luminal A.

**
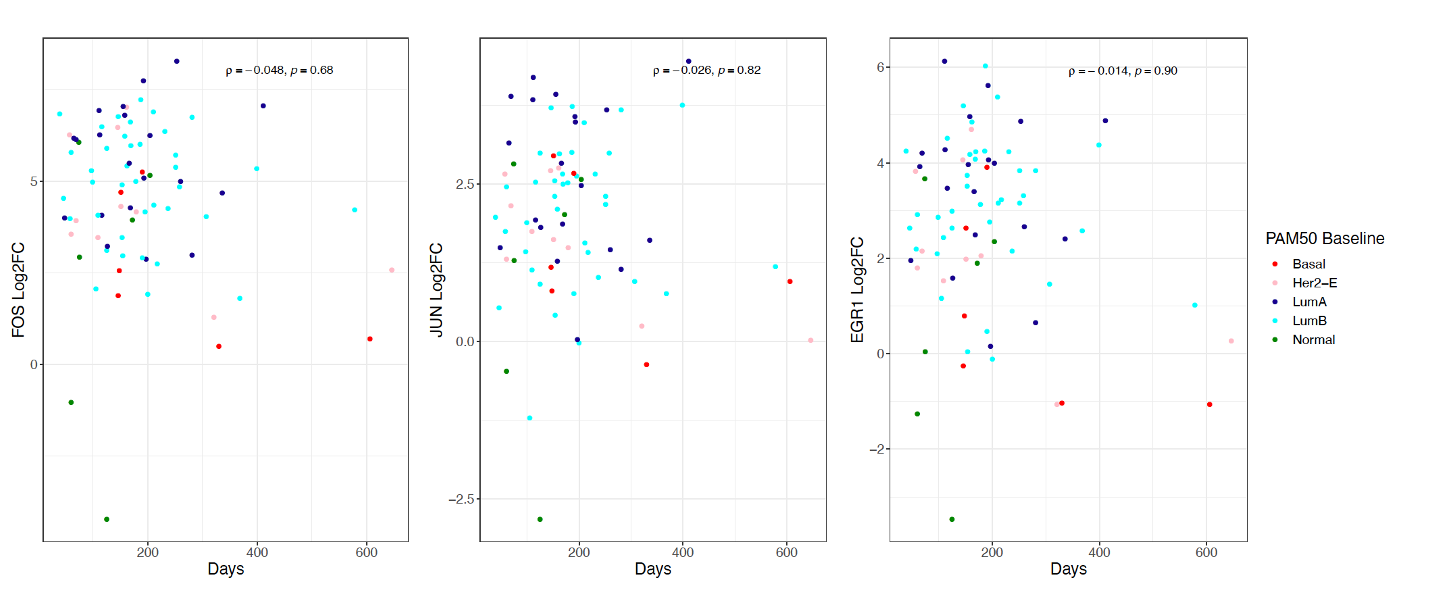
**


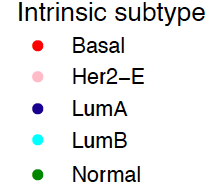


**Supplementary figure S5**. A. Unsupervised hierarchical clustering of module scores at baseline in the POETIC treated subset selected by two unpaired SAM analysis for Ki67 H-H vs H-L categories and median centered. **B.** Significant modules using two unpaired SAM analysis of the differential changes in module scores between H-H vs H-L in POETIC treated samples. **Abbreviations**: H-H: Ki67 High_baseline_- Ki67 High_surgery,_ H-L: Ki67 High_baseline_- Ki67 Low_surgery,_ Her2-E: Her2 enriched, LumB: Luminal B, LumA: Luminal A, 2wk: 2 weeks timepoint, GE: Gene expression


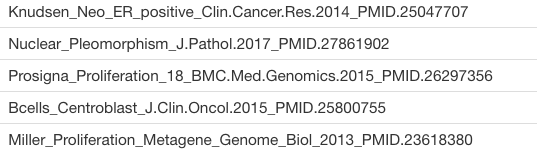
**
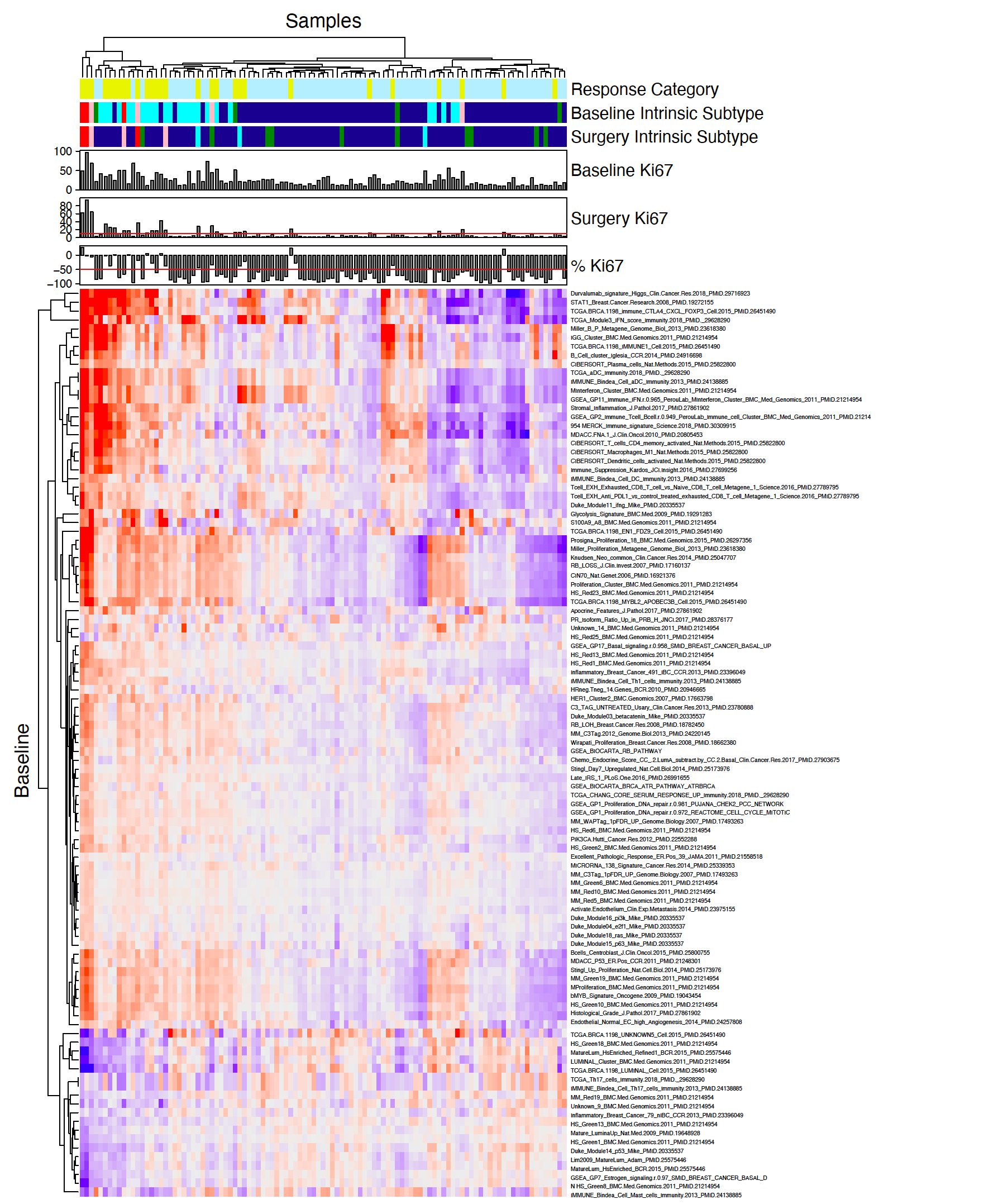
A. B.**

**
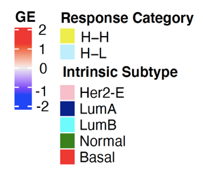
**

Immune-related modules

Proliferation-related modules

ER signalling and other pathways

**Supplementary figure S6.** Unsupervised hierarchical clustering of single gene expression changes in the NeoAI subset selected by two unpaired SAM analysis for Ki67 H-H vs H-L. **Abbreviations**: H-H: Ki67 High_baseline_- Ki67 High_surgery,_ H-L: Ki67 High_baseline_- Ki67 Low_surgery,_ Her2-E: Her2 enriched, LumB: Luminal B, LumA: Luminal A, 2wk: 2 weeks timepoint, GE: Gene expression.

**
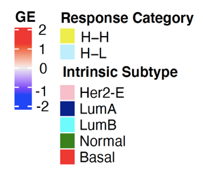

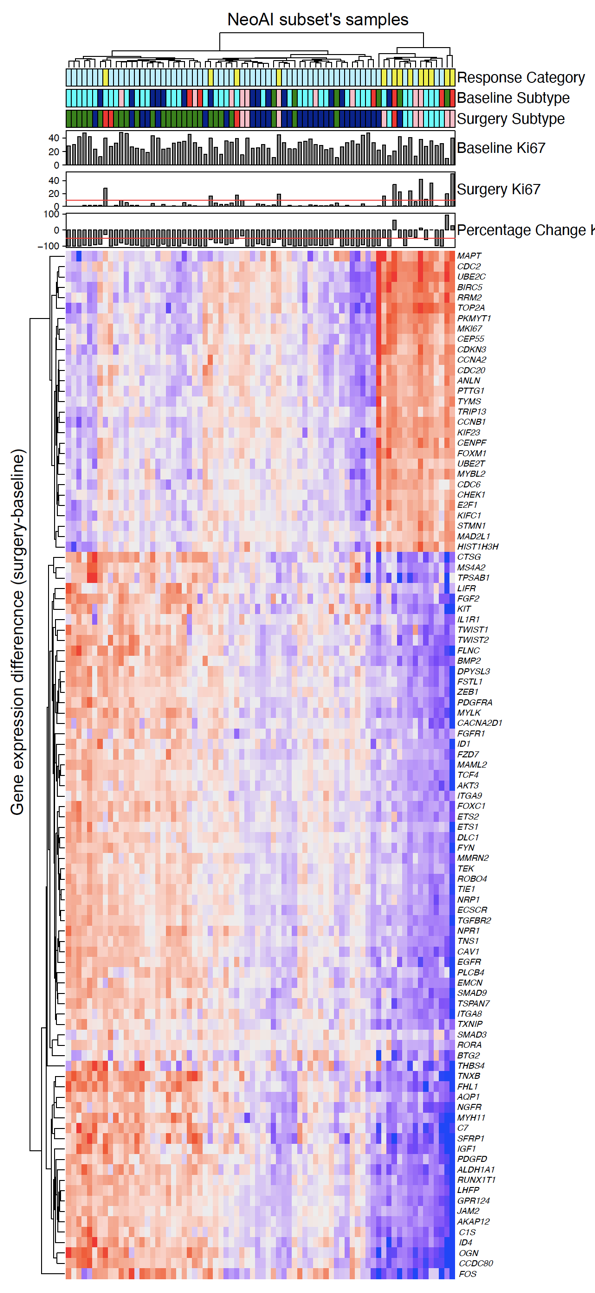
**

**Supplementary figure S7**. Boxplots showing gene signature expression of the two immune-related module-score (”Durvalumab” and “Immune-tolerance”) at baseline and at surgery amongst H-H and H-L Ki67 response categories in A. POETIC and B. NeoAI; C. Differential changes in gene expression for each individual gene included in the two immune-related modules: “Durvalumab” and “Immune-tolerance” between H-H and H-L tumours in the NeoAI subset. **Abbreviations**: H-H: Ki67 High_baseline_- Ki67 High_surgery,_ H-L: Ki67 High_baseline_- Ki67 Low_surgery,_ Her2-E: Her2 enriched, LumB: Luminal B, LumA: Luminal A, 2wk: 2 weeks timepoint


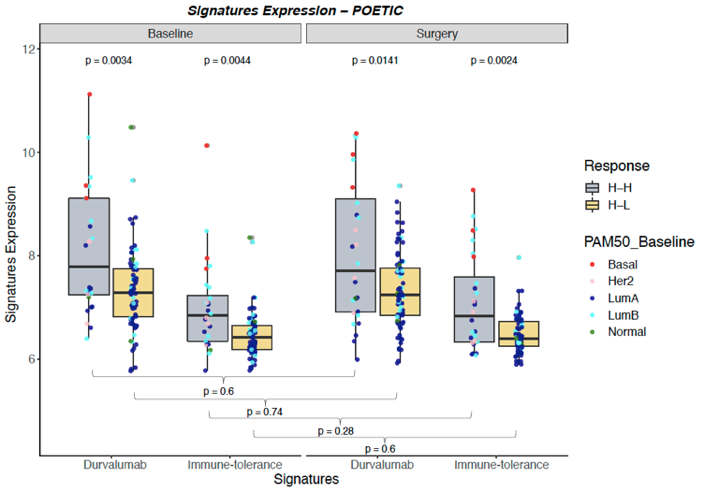
**
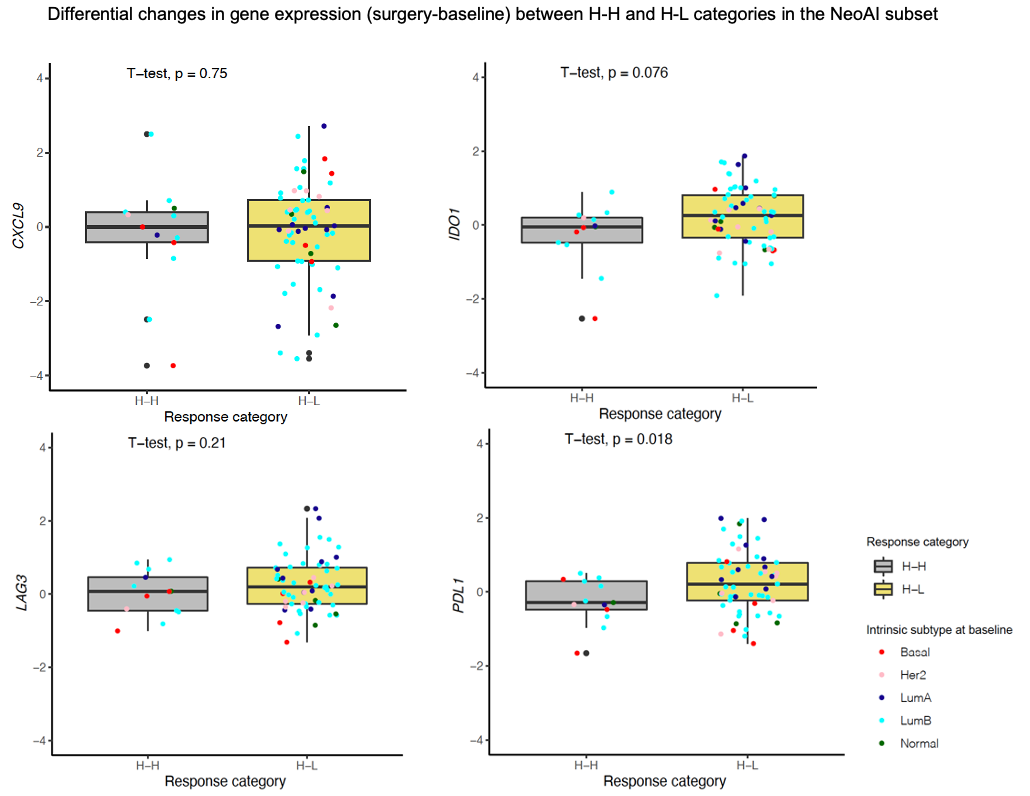
A. C.**

**
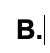
**


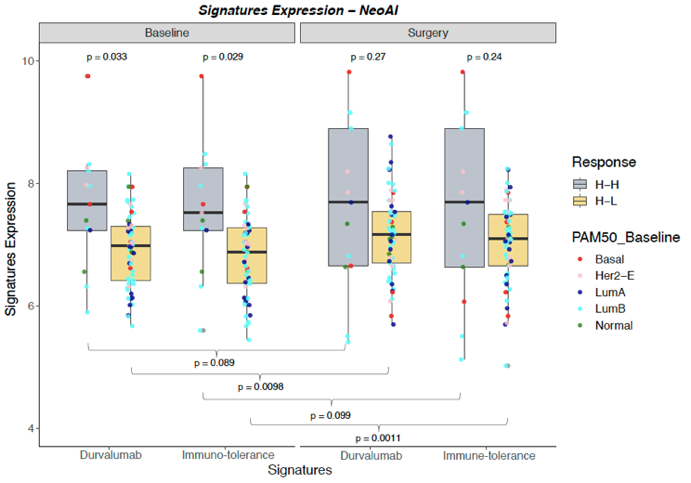


.

**Supplementary figure S8.** Spearman rank order correlation of the changes in gene expression from baseline to surgery (Log2FC) under AI treatment of the two immune related signatures “Durvalumab” and “Immune-tolerance” with time in the NeoAI study.

**Abbreviations**: FC: Fold change, Her2-E: Her2-enriched, LumB: Luminal B, LumA: Luminal A.


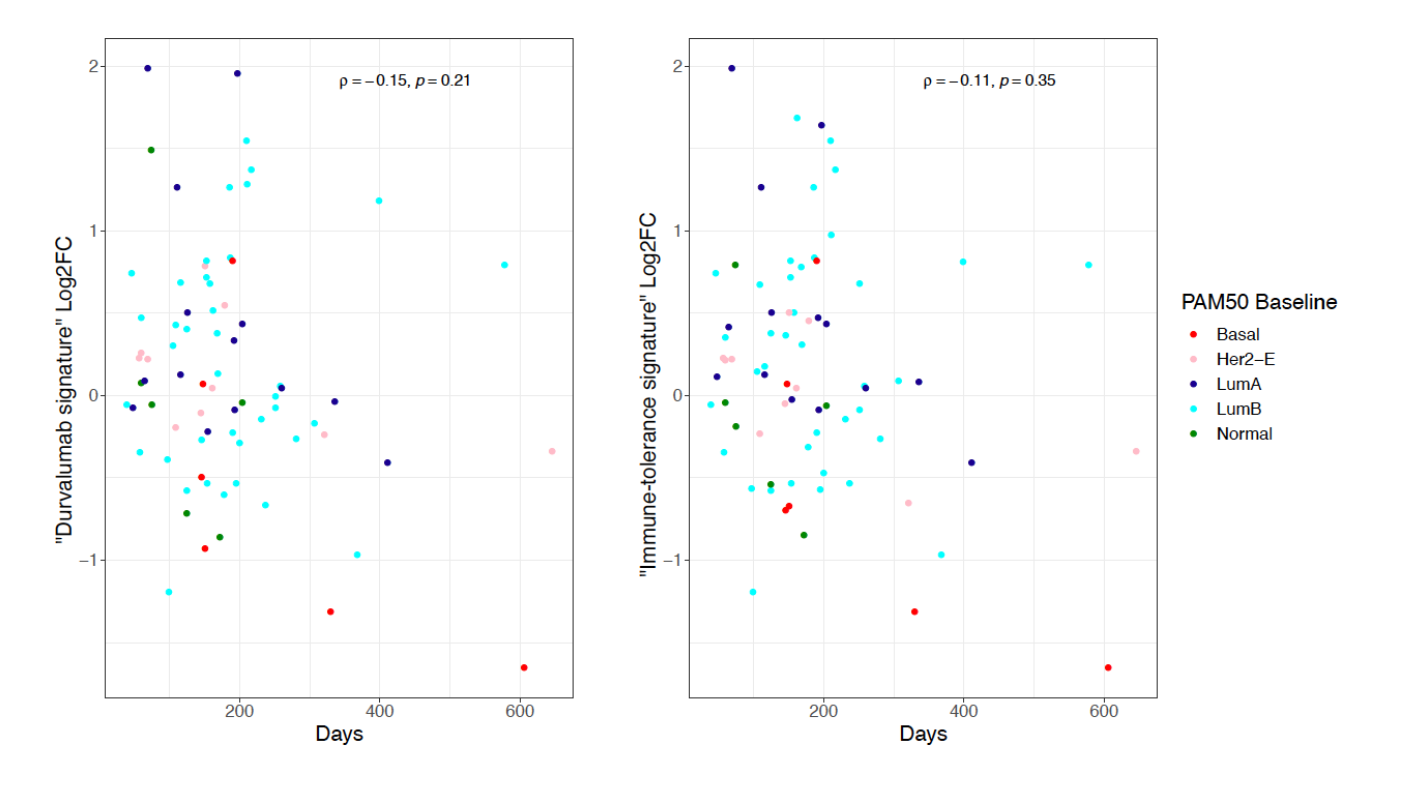


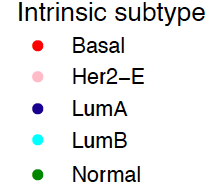

Supplement: Supplementary Figure [file ccr-21-2718_supplementary_figures_supp1.docx]
