# Supplementary material for: Impact of Duration of Neoadjuvant Aromatase Inhibitors on Molecular Expression Profiles in Estrogen Receptor–positive Breast Cancers
Source: Clin Cancer Res. 2022 Mar 14;28(6):1217–28. doi: 10.1158/1078-0432.CCR-21-2718 (PMC7612503; doi:10.1158/1078-0432.CCR-21-2718)
Supplement: Supplementary Data [file ccr-21-2718_supplementary_tables_supp2.docx]

**Supplementary table S1.** Demographics and molecular characteristics in POETIC subset (treatment and control arms separately) and in the NeoAI study. **Abbreviations:** H-H: Ki67 High_baseline_- Ki67 High_surgery,_ H-L: Ki67 High_baseline_- Ki67 Low_surgery,_ L-L: Ki67 Low_baseline_- Ki67 Low_surgery_, L-H: Ki67 Low_baseline_-High_surgery_, Her2-E: Her2 enriched, LumB: Luminal B, LumA: Luminal A.

|  | POETIC (treatment arm) | POETIC (control arm) | NeoAI study |
| --- | --- | --- | --- |
|  | **n=137** | **n=47** | **n=80** |
| **Surgery Tumour size (cm)** | | | |
| ≤2 | 39 (28.5%) | 13 (27.7%) | 23 (28.8%) |
| >2 & ≤5 | 94 (68.6%) | 29 (61.7%) | 46 (57.5%) |
| >5 | 4 (2.90%) | 5 (10.6%) | 11 (13.7%) |
| **Nodal status** | | | |
| Negative | 69 (50.4%) | 27 (57.4%) | 43 (53.8%) |
| Positive | 68 (49.6%) | 20 (42.6%) | 22 (27.5%) |
| NA | 0 (0.0%) | 0 (0.00%) | 15 (18.7%) |
| **Histological type** | | | |
| Ductal | 109 (79.6%) | 38 (80.9%) | 60 (75.0%) |
| Lobular | 25 (18.2%) | 9 (19.1%) | 12 (15.0%) |
| Mixed ductal and lobular | 0 (0.0%) | 0 (0.0%) | 8 (10.0%) |
| Mucinous | 1 (0.73%) | 1 (0.0%) | 0 (0.0%) |
| Papillary | 1 (0.73%) | 2 (0.0%) | 1 (0.0%) |
| Tubular | 1 (0.73%) | 3 (0.0%) | 2 (0.0%) |
| **HER2 status** | | | |
| Negative | 118 (86.1%) | 39 (82.9%) | 75 (93.8%) |
| Positive | 19 (13.9%) | 8 (7.10%) | 5 (6.2%) |
| **PAM50 subtype at baseline** | | | |
| Basal Like | 3 (2.2%) | 0 (0%) | 6 (7.5%) |
| Her2-E | 12 (8.80%) | 1 (2.10%) | 10 (12.5%) |
| LumA | 88 (64.2%) | 33 (70.2%) | 20 (25.0%) |
| LumB | 30 (21.9%) | 12 (25.5%) | 38 (47.5%) |
| Normal | 4 (2.9%) | 1 (2.1%) | 6 (7.5%) |
| **Change of subtype** | | | |
| Yes | 52 (38.0%) | 11 (23.4%) | 54 (67.5%) |
| No | 85 (62.0%) | 36 (76.6%) | 26 (32.5%) |
| **Ki67 changes** | | | |
| H-H | 42 (30.7%) | 34 (72.3%) | 13 (16.3%) |
| H-L | 82 (59.8%) | 6 (12.8%) | 61 (76.3%) |
| L-H | 0 (0.0%) | 6 (12.8%) | 1 (1.2%) |
| L-L | 13 (9.5%) | 1 (2.1%) | 5 (6.2%) |

**Supplementary table S2.** Number of genes significantly differentially expressed between baseline and surgery in each of the two cohorts according to different subgroups within each cohort.

|  | POETIC subset | NeoAI study |
| --- | --- | --- |
| All tumours | 21 genes | 54 genes |
| Luminal tumours | 26 genes | 100 genes |
| Luminal A | 27 genes | 77 genes |
| Luminal B | 54 genes | 109 genes |
| Controls | 8 genes |  |

**Supplementary table S3**. Significant differential changes for single gene level between baseline and surgery in the NeoAI study and in POETIC subset according to treatment arm and ranked by Log2FC values. **Abbreviations:** ID: Identification, Log2FC: Log2 Fold Change, FDR: False Discovery Rate.

| ***NeoAI*** | | | ***POETIC (treated)*** | | | ***POETIC (controls)*** | | |
| --- | --- | --- | --- | --- | --- | --- | --- | --- |
| ***Gene ID*** | **Log2FC** | **p-adjusted value (FDR)** | ***Gene ID*** | **Log2FC** | **p-adjusted value (FDR)** | ***Gene ID*** | **Log2FC** | **p-adjusted value (FDR)** |
| ***Inhibited*** | | | | | | | | |
| ***PGR*** | -1.4 | <0.0001 | ***TFF1*** | -1.6 | <0.0001 | ***HBB*** | -1.6 | 0.01 |
| ***TOP2A*** | -1.3 | <0.0001 | ***TOP2A*** | -1.4 | <0.0001 | ***HBA2*** | -1.5 | 0.01 |
| ***BIRC5*** | -1.0 | <0.0001 | ***UBE2C*** | -1.4 | <0.0001 | ***HBA1*** | -1.3 | 0.017 |
| ***PKMYT1*** | -1.6 | <0.0001 | ***HBB*** | -1.3 | <0.0001 | ***RNY5*** | -1.2 | 0.022 |
| ***MAPT*** | -1.2 | <0.0001 | ***HBA2*** | -1.3 | <0.0001 |  |  |  |
| ***UBE2C*** | -1.8 | <0.0001 | ***CDC20*** | -1.2 | <0.0001 |  |  |  |
| ***RRM2*** | -1.4 | <0.0001 | ***NUSAP1*** | -1.2 | <0.0001 |  |  |  |
| ***MKI67*** | -1.8 | <0.0001 | ***HBA1*** | -1.1 | <0.0001 |  |  |  |
| ***CENPF*** | -1.2 | <0.0001 | ***NEK2*** | -1.1 | <0.0001 |  |  |  |
| ***KIF23*** | -1.3 | <0.0001 | ***SUSD3*** | -1.1 | <0.0001 |  |  |  |
| ***CDC2*** | -1.9 | <0.0001 | ***ASPM*** | -1.0 | <0.0001 |  |  |  |
| ***NAT1*** | -1.2 | <0.0001 | ***UHRF1*** | -1.0 | <0.0001 |  |  |  |
| ***ANLN*** | -1.5 | <0.0001 | ***PRC1*** | -1.0 | <0.0001 |  |  |  |
| ***MYBL2*** | -1.1 | <0.0001 | ***FGFR3*** | -1.0 | <0.0001 |  |  |  |
| ***KIFC1*** | -1.2 | <0.0001 | ***AGR2*** | -1.0 | <0.0001 |  |  |  |
| ***CCNB1*** | -1.1 | <0.0001 |  |  |  |  |  |  |
| ***MMP11*** | -1.6 | <0.0001 |  |  |  |  |  |  |
| ***CEP55*** | -1.5 | <0.0001 |  |  |  |  |  |  |
| ***CDC20*** | -1.3 | <0.0001 |  |  |  |  |  |  |
| ***CCND1*** | -1.3 | <0.0001 |  |  |  |  |  |  |
| ***TRIP13*** | -1.2 | <0.0001 |  |  |  |  |  |  |
| **Activated** | | | | | | | | |
| **FOS** | 4.6 | <0.0001 | ***FOS*** | 2.2 | <0.0001 | ***FOS*** | 1.83 | 0.00051 |
| **EGR1** | 2.0 | <0.0001 | ***RGS1*** | 1.8 | <0.0001 | ***RGS1*** | 1.55 | 0.0012 |
| **NR4A1** | 2.7 | <0.0001 | ***DUSP1*** | 1.8 | <0.0001 | ***DUSP1*** | 1.54 | 0.00027 |
| **OGN** | 2.7 | <0.0001 | ***FOSB*** | 1.5 | <0.0001 | ***FOSB*** | 1.12 | 0.021 |
| **JUN** | 1.3 | <0.0001 | ***CYR61*** | 1.3 | <0.0001 |  |  |  |
| **NR4A3** | 2.0 | <0.0001 | ***EGR1*** | 1.3 | <0.0001 |  |  |  |
| **CCDC80** | 1.2 | <0.0001 |  |  |  |  |  |  |
| **IL6** | 1.2 | <0.0001 |  |  |  |  |  |  |
| **CTSG** | 1.5 | <0.0001 |  |  |  |  |  |  |
| **C7** | 1.1 | <0.0001 |  |  |  |  |  |  |
| **BTG2** | 1.2 | <0.0001 |  |  |  |  |  |  |
| **ID4** | 1.0 | <0.0001 |  |  |  |  |  |  |
| **SFRP1** | 1.2 | <0.0001 |  |  |  |  |  |  |
| **KRT14** | 1.6 | <0.0001 |  |  |  |  |  |  |
| **IGF1** | 1.6 | <0.0001 |  |  |  |  |  |  |
| **COL3A1** | 1.4 | <0.0001 |  |  |  |  |  |  |
| **TNXB** | 1.2 | <0.0001 |  |  |  |  |  |  |
| **GAS1** | 1.2 | <0.0001 |  |  |  |  |  |  |
| **DCN** | 1.3 | <0.0001 |  |  |  |  |  |  |
| **PDGFD** | 1.3 | <0.0001 |  |  |  |  |  |  |
| **TWIST1** | 1.1 | <0.0001 |  |  |  |  |  |  |
| **CCL4** | 1.4 | <0.0001 |  |  |  |  |  |  |
| **AKAP12** | 1.2 | <0.0001 |  |  |  |  |  |  |
| **CMA1** | 1.3 | <0.0001 |  |  |  |  |  |  |
| **ALDH1A1** | 1.1 | <0.0001 |  |  |  |  |  |  |
| **FHL1** | 1.1 | <0.0001 |  |  |  |  |  |  |
| **SOCS3** | 1.0 | <0.0001 |  |  |  |  |  |  |
| **TSPAN7** | 1.2 | <0.0001 |  |  |  |  |  |  |
| **MMP2** | 1.3 | <0.0001 |  |  |  |  |  |  |
| **CXCL12** | 1.1 | <0.0001 |  |  |  |  |  |  |
| **C1S** | 1.3 | <0.0001 |  |  |  |  |  |  |
| **SMAD9** | 1.1 | <0.0001 |  |  |  |  |  |  |
| **NGFR** | 1.3 | <0.0001 |  |  |  |  |  |  |

**Supplementary table S4.** Multivariable cox regression models for TTR and OS for each of the significant findings in the POETIC subset: in blue changes in the correlation coefficient scores to prototypical intrinsic subtype centroids; in orange changes of the modules scores that were significantly different from baseline to surgery in all tumours including Luminal B, in green those significant changes from baseline to surgery in Luminal B only, in grey those changes in modules score observed significant differentially expressed between H-H and H-L tumours by SAM analysis (light grey also includes modules significantly different expressed between baseline and surgery in Luminal B tumours and dark green in Luminal B and in all tumours). The multivariable cox models were adjusted for the standard clinicopathological variables: PR status, HER2 status, tumour grade,pathological tumour size, histology subtype, nodal status, and vascular invasion. **Abbreviations:** TTR: Time to Recurrence, OS: Overall Survival, CI: Confidence Interval, FDR: False discovery rate, Her2-E: Her2 enriched, LumB: Luminal B, LumA: Luminal A, 2wk: 2 weeks timepoint, H-H: Ki67 High_baseline_- Ki67 High_surgery,_ H-L: Ki67 High_baseline_- Ki67 Low_surgery,_

|  | **Multivariable cox models for TTR** | | | | | **Multivariable cox models for OS** | | | | |
| --- | --- | --- | --- | --- | --- | --- | --- | --- | --- | --- |
|  | **Hazard Ratio** | **95% CI** | | **p-value** | **FDR** | **Hazard Ratio** | **95% CI** | | **p-value** | **FDR** |
| Basal_centroid_change | 3.81 | 0.16 | 91.45 |  |  | 0.07 | 0.01 | 0.82 | ** | * |
| Her2E_centroid_change | 4.12 | 0.12 | 138.19 |  |  | 1.42 | 0.08 | 24.21 |  |  |
| LumA_centroid_change | 0.11 | 0.01 | 1.04 | * | * | 1.21 | 0.2 | 7.2 |  |  |
| LumB_centroid_change | 10.66 | 1.03 | 110.8 | ** | * | 12.18 | 1.74 | 85.47 | ** | * |
| Normal_centroid_change | 0.1 | 0.01 | 0.72 | ** | ** | 0.22 | 0.04 | 1.14 | * |  |
| TCGABRCA1198_MYBL2_APOBEC3B_Ce | 12.23 | 3.51 | 42.54 | *** | *** | 2.38 | 0.89 | 6.37 | * |  |
| Proliferation_Cluster_BMCMedGe | 12.19 | 3.36 | 44.26 | *** | *** | 3.22 | 1.07 | 9.66 | ** | * |
| CIN70_NatGenet2006_PMID169213 | 15.78 | 3.99 | 62.36 | *** | *** | 3.81 | 1.18 | 12.26 | ** | * |
| RB_LOSS_JClinInvest2007_PMID | 13.61 | 3.47 | 53.39 | *** | *** | 3.21 | 1.03 | 10 | ** |  |
| HS_Red23_BMCMedGenomics2011_P | 14 | 3.55 | 55.17 | *** | *** | 3.13 | 0.98 | 9.98 | * |  |
| Knudsen_Neo_common_ClinCancerR | 15.31 | 3.65 | 64.26 | *** | *** | 5.54 | 1.67 | 18.37 | *** | * |
| MProliferation_BMCMedGenomics | 18.63 | 3.93 | 88.25 | *** | *** | 2.9 | 0.81 | 10.39 |  |  |
| bMYB_Signature_Oncogene2009_PMI | 25.28 | 4.92 | 129.83 | *** | *** | 3.9 | 0.97 | 15.64 | * |  |
| TCGABRCA1198_immune_FOS_JUN_IL | 0.32 | 0.15 | 0.67 | *** | *** | 0.53 | 0.3 | 0.94 | ** | * |
| FOS_JUN_Cluster_BMCMedGenomics | 0.31 | 0.15 | 0.65 | *** | *** | 0.59 | 0.34 | 1.02 | * |  |
| Histological_Grade_JPathol2017 | 32.64 | 4.94 | 215.45 | *** | *** | 6.16 | 1.24 | 30.51 | ** | * |
| Stingl_Up_Proliferation_NatCell | 27.03 | 4.7 | 155.49 | *** | *** | 5.47 | 1.25 | 23.98 | ** | * |
| MDACC_P53_ERPos_CCR2011_PMID2 | 26.36 | 4.06 | 171.29 | *** | *** | 3.32 | 0.77 | 14.34 |  |  |
| MM_Green19_BMCMedGenomics2011 | 53.55 | 6.56 | 437.33 | *** | *** | 7.23 | 1.3 | 40.22 | ** | * |
| HS_Green10_BMCMedGenomics2011 | 48.32 | 6.45 | 362.14 | *** | *** | 3.23 | 0.56 | 18.71 |  |  |
| Chemo_Endocrine_Score_CC_2LumA | 28.65 | 3.59 | 228.56 | *** | *** | 5.84 | 0.93 | 36.73 | * |  |
| MET_DOWN_Significant_Genes_LOW_B | 0.57 | 0.2 | 1.59 |  |  | 0.58 | 0.27 | 1.26 |  |  |
| HS_Green9_BMCMedGenomics2011_ | 0.67 | 0.23 | 1.95 |  |  | 0.57 | 0.25 | 1.28 |  |  |
| HS_Red21_BMCMedGenomics2011_P | 0.67 | 0.25 | 1.79 |  |  | 0.58 | 0.28 | 1.21 |  |  |
| TCGABRCA1198_COLLAGEN11A_Cell | 0.66 | 0.3 | 1.47 |  |  | 0.59 | 0.32 | 1.09 | * |  |
| MDACCFNA2_JClinOncol2010_PM | 0.5 | 0.16 | 1.59 |  |  | 0.48 | 0.2 | 1.2 |  |  |
| Fibroblast_Cluster_BMCMedGenom | 0.59 | 0.23 | 1.52 |  |  | 0.55* | 0.27 | 1.1 | * |  |
| Nuclear_Pleomorphism_JPathol20 | 32.76 | 3.24 | 331.64 | *** | *** | 9.37 | 1.36 | 64.79 | ** | * |
| Bcells_Centroblast_JClinOncol | 21.27 | 3.77 | 119.83 | *** | *** | 2.05 | 0.5 | 8.42 |  |  |
| Prosigna_Proliferation_18_BMCMe | 7.07 | 2.62 | 19.08 | *** | *** | 2.38 | 1.06 | 5.39 | ** | * |
| Knudsen_Neo_ER_positive_ClinCan | 23.14 | 4.76 | 112.49 | *** | *** | 8.05 | 2.06 | 31.47 | *** | * |
| Miller_Proliferation_Metagene_Ge | 6.6 | 2.53 | 17.23 | *** | *** | 2.28 | 1.02 | 5.1 | ** |  |

*** p<0.01, ** p<0.05, * p<0.1

**Supplementary table S5.** Multivariable cox regression models for Overall survival (OS) and multivariable linear regression models for risk of recurrence score (ROR) as surrogate of recurrence in the NeoAI subset: in blue changes in the correlation coefficient scores to prototypical intrinsic subtype centroids; in orange changes of the genes that were significantly different from baseline to surgery in all tumours including Luminal B; in green those significant changes in Luminal B only, in yellow, grey and purple the significantly different changes of gene expression between H-H and H-L tumours: in grey there are genes that also changed significantly from baseline to surgery in luminal B only and in purple in all patients. The models have been adjusted for the standard post-surgery clinicopathological variables: PR status, HER2 status, diagnostic-tumour grade, surgical tumour size, diagnostic histological type, nodal status, and vascular invasion. **Abbreviations**: OS: Overall Survival, ROR: Risk of Recurrence Score, CI: Confidence Interval, FDR: False discovery rate, Her2-E: Her2 enriched, LumB: Luminal B, LumA: Luminal A, 2wk: 2 weeks timepoint, H-H: Ki67 High_baseline_- Ki67 High_surgery,_ H-L: Ki67 High_baseline_- Ki67 Low_surgery,_

|  | **Adjusted cox models for OS** | | | | | **Adjusted linear regression models for ROR at surgery** | | |
| --- | --- | --- | --- | --- | --- | --- | --- | --- |
|  |  | **95% CI** | |  |  |  |  |  |
|  | Hazard Ratio | 5% | 95% | p-value | FDR | Correlation Coefficient | p-value | FDR |
| Basal_centroid_change | 0.20 | 0.03 | 1.44 |  |  | -0.25 |  |  |
| Her2_centroid_change | 3.76 | 0.88 | 16.06 |  |  | 0.69 | *** | *** |
| LumA_centroid_change | 0.63 | 0.14 | 2.79 |  |  | -0.49 | *** | *** |
| LumB_centroid_change | 3.60 | 1.25 | 10.35 | ** |  | 0.68 | *** | *** |
| Normal_centroid_change | 0.33 | 0.14 | 0.81 | ** |  | -0.71 | *** | *** |
| *CCL4* | 1.01 | 0.70 | 1.45 |  |  | -0.39 |  |  |
| *CMA1* | 0.75 | 0.53 | 1.07 |  |  | -0.52 | *** | *** |
| *COL3A1* | 0.68 | 0.49 | 0.93 | ** |  | -0.59 | *** | *** |
| *CXCL12* | 0.63 | 0.45 | 0.89 | ** | * | -0.57 | *** | *** |
| *DCN* | 0.61 | 0.45 | 0.82 | *** | ** | -0.66 | *** | *** |
| *EGR1* | 0.94 | 0.78 | 1.15 |  |  | -0.60 | *** | *** |
| *GAS1* | 0.68 | 0.49 | 0.95 | * |  | -0.61 | *** | *** |
| *IL6* | 0.81 | 0.60 | 1.11 |  |  | -0.47 | *** | *** |
| *JUN* | 0.87 | 0.66 | 1.14 |  |  | -0.61 | *** | *** |
| *KRT14* | 0.82 | 0.71 | 0.95 | ** | * | -0.48 | *** | *** |
| *MMP11* | 1.22 | 0.91 | 1.63 |  |  | 0.09 |  |  |
| *MMP2* | 0.75 | 0.53 | 1.05 |  |  | -0.59 | *** | *** |
| *NAT1* | 1.32 | 0.98 | 1.78 |  |  | 0.40 | *** | ** |
| *NR4A1* | 0.91 | 0.76 | 1.10 |  |  | -0.51 | *** | *** |
| *NR4A3* | 0.95 | 0.74 | 1.22 |  |  | -0.42 | ** | ** |
| *PGR* | 1.02 | 0.80 | 1.31 |  |  | 0.31 |  |  |
| *SOCS3* | 0.83 | 0.66 | 1.04 |  |  | -0.45 | *** | *** |
| *ACTG2* | 0.61 | 0.44 | 0.84 | ** | * | -0.58 | *** | *** |
| *C1R* | 0.70 | 0.51 | 0.96 | * |  | -0.63 | *** | *** |
| *CCL19* | 0.83 | 0.62 | 1.11 |  |  | -0.19 |  |  |
| *CCND1* | 1.00 | 0.65 | 1.55 |  |  | 0.43 | *** | *** |
| *CCNE2* | 1.72 | 1.12 | 2.65 | ** | * | 0.47 | *** | *** |
| *CDCA1* | 1.30 | 0.79 | 2.13 |  |  | 0.47 | *** | *** |
| *CHIT1* | 1.10 | 0.80 | 1.50 |  |  | -0.19 |  |  |
| *CNN1* | 0.53 | 0.36 | 0.78 | *** | ** | -0.58 | *** | *** |
| *COL1A1* | 0.75 | 0.55 | 1.04 |  |  | -0.48 | *** | *** |
| *COL1A2* | 0.71 | 0.52 | 0.96 | * |  | -0.58 | *** | *** |
| *COL6A6* | 0.64 | 0.44 | 0.93 | ** |  | -0.40 | *** | *** |
| *DPP4* | 0.90 | 0.62 | 1.31 |  |  | -0.50 | *** | *** |
| *DUSP6* | 0.90 | 0.55 | 1.46 |  |  | -0.45 | *** | *** |
| *ECM2* | 0.56 | 0.39 | 0.82 | ** | * | -0.61 | *** | *** |
| *FGF7* | 0.53 | 0.35 | 0.80 | ** | * | -0.53 | *** | *** |
| *FIGF* | 0.75 | 0.52 | 1.07 |  |  | -0.47 | *** | *** |
| *GSN* | 0.54 | 0.38 | 0.78 | *** | ** | -0.64 | *** | *** |
| *HELLS* | 1.79 | 0.96 | 3.33 |  |  | 0.46 | *** | *** |
| *IGF1R* | 1.37 | 0.77 | 2.42 |  |  | -0.03 |  |  |
| *KRT17* | 0.79 | 0.64 | 0.96 | ** |  | -0.54 | *** | *** |
| *KRT5* | 0.83 | 0.68 | 1.01 |  |  | -0.46 | *** | *** |
| *MCM4* | 1.50 | 0.85 | 2.66 |  |  | 0.56 | *** | *** |
| *MET* | 0.65 | 0.42 | 1.00 | * |  | -0.52 | *** | *** |
| *MS4A1* | 0.84 | 0.61 | 1.15 |  |  | -0.28 |  |  |
| *NEFL* | 0.91 | 0.61 | 1.35 |  |  | -0.38 | ** | ** |
| *NTRK2* | 0.78 | 0.58 | 1.06 |  |  | -0.32 | * | * |
| *PROM1* | 0.81 | 0.59 | 1.12 |  |  | -0.37 | * | * |
| *TNFAIP3* | 1.05 | 0.68 | 1.61 |  |  | -0.34 | ** | ** |
| *WIF1* | 0.73 | 0.52 | 1.02 |  |  | -0.45 | *** | *** |
| *WNT11* | 1.22 | 0.75 | 1.99 |  |  | -0.37 | ** | ** |
| *ZBTB16* | 0.97 | 0.75 | 1.25 |  |  | -0.38 | *** | *** |
| *AKT3* | 0.42 | 0.25 | 0.69 | *** | ** | -0.71 | *** | *** |
| *CACNA2D1* | 0.45 | 0.30 | 0.68 | *** | ** | -0.55 | *** | *** |
| *CDKN3* | 1.27 | 0.89 | 1.81 |  |  | 0.69 | *** | *** |
| *CHEK1* | 1.78 | 0.98 | 3.21 |  |  | 0.67 | *** | *** |
| *DLC1* | 0.54 | 0.34 | 0.86 | ** | * | -0.55 | *** | *** |
| *DPYSL3* | 0.42 | 0.29 | 0.62 | *** | ** | -0.61 | *** | *** |
| *ECSCR* | 0.43 | 0.27 | 0.68 | *** | ** | -0.58 | *** | *** |
| *EMCN* | 0.52 | 0.34 | 0.80 | ** | * | -0.59 | *** | *** |
| *ETS1* | 0.27 | 0.15 | 0.47 | *** | ** | -0.50 | *** | *** |
| *ETS2* | 0.59 | 0.37 | 0.94 | * |  | -0.54 | *** | *** |
| *FGFR1* | 0.46 | 0.25 | 0.83 | ** | * | -0.65 | *** | *** |
| *FOS* | 0.93 | 0.79 | 1.09 |  |  | -0.57 | *** | *** |
| *FYN* | 0.37 | 0.23 | 0.61 | *** | ** | -0.56 | *** | *** |
| *FZD7* | 0.38 | 0.22 | 0.68 | *** | ** | -0.64 | *** | *** |
| *GPR124* | 0.54 | 0.38 | 0.78 | *** | ** | -0.71 | *** | *** |
| *ID1* | 0.45 | 0.28 | 0.72 | *** | ** | -0.58 | *** | *** |
| *IL1R1* | 0.75 | 0.49 | 1.16 |  |  | -0.42 | *** | *** |
| *ITGA8* | 0.55 | 0.35 | 0.86 | ** | * | -0.58 | *** | *** |
| *ITGA9* | 0.45 | 0.26 | 0.79 | ** | * | -0.61 | *** | *** |
| *KIFC1* | 1.22 | 0.79 | 1.87 |  |  | 0.58 | *** | *** |
| *LIFR* | 0.55 | 0.33 | 0.92 | * |  | -0.49 | *** | *** |
| *MAD2L1* | 2.69 | 1.31 | 5.50 | ** | * | 0.56 | *** | *** |
| *MAML2* | 0.42 | 0.25 | 0.70 | *** | ** | -0.63 | *** | *** |
| *MMRN2* | 0.43 | 0.25 | 0.74 | *** | ** | -0.64 | *** | *** |
| *NPR1* | 0.47 | 0.31 | 0.72 | *** | ** | -0.53 | *** | *** |
| *NRP1* | 0.44 | 0.27 | 0.72 | *** | ** | -0.60 | *** | *** |
| *PDGFRA* | 0.47 | 0.29 | 0.74 | *** | ** | -0.62 | *** | *** |
| *PLCB4* | 0.64 | 0.40 | 1.02 |  |  | -0.55 | *** | *** |
| *ROBO4* | 0.42 | 0.26 | 0.69 | *** | ** | -0.57 | *** | *** |
| *RORA* | 0.83 | 0.48 | 1.43 |  |  | -0.54 | *** | *** |
| *RUNX1T1* | 0.68 | 0.47 | 1.00 | * |  | -0.66 | *** | *** |
| *SMAD3* | 0.78 | 0.37 | 1.64 |  |  | -0.32 | * | * |
| *STMN1* | 2.02 | 1.18 | 3.47 | ** | * | 0.63 | *** | *** |
| *TCF4* | 0.56 | 0.33 | 0.95 | * |  | -0.65 | *** | *** |
| *TEK* | 0.41 | 0.23 | 0.72 | *** | ** | -0.56 | *** | *** |
| *TGFBR2* | 0.41 | 0.26 | 0.67 | *** | ** | -0.63 | *** | *** |
| *THBS4* | 0.82 | 0.63 | 1.07 |  |  | -0.43 | *** | *** |
| *TIE1* | 0.44 | 0.27 | 0.70 | *** | ** | -0.63 | *** | *** |
| *TNS1* | 0.39 | 0.23 | 0.67 | *** | ** | -0.68 | *** | *** |
| *TPSAB1* | 0.89 | 0.68 | 1.17 |  |  | -0.47 | *** | *** |
| *UBE2T* | 1.41 | 0.85 | 2.33 |  |  | 0.60 | *** | *** |
| *ZEB1* | 0.48 | 0.32 | 0.73 | *** | ** | -0.59 | *** | *** |
| *AQP1* | 0.37 | 0.23 | 0.58 | *** | ** | -0.59 | *** | *** |
| *BMP2* | 0.78 | 0.56 | 1.10 |  |  | -0.56 | *** | *** |
| *CAV1* | 0.52 | 0.33 | 0.81 | ** | * | -0.60 | *** | *** |
| *CCNA2* | 1.08 | 0.68 | 1.73 |  |  | 0.69 | *** | *** |
| *CDC6* | 1.29 | 0.78 | 2.12 |  |  | 0.60 | *** | *** |
| *E2F1* | 1.56 | 0.99 | 2.48 |  |  | 0.65 | *** | *** |
| *EGFR* | 0.68 | 0.46 | 0.99 | * |  | -0.55 | *** | *** |
| *FGF2* | 0.58 | 0.39 | 0.86 | ** | * | -0.53 | *** | *** |
| *FLNC* | 0.57 | 0.40 | 0.82 | ** | * | -0.60 | *** | *** |
| *FOXC1* | 0.67 | 0.42 | 1.06 |  |  | -0.59 | *** | *** |
| *FOXM1* | 1.61 | 1.05 | 2.45 | * |  | 0.66 | *** | *** |
| *FSTL1* | 0.54 | 0.36 | 0.82 | ** | * | -0.57 | *** | *** |
| *HIST1H3H* | 1.26 | 0.84 | 1.89 |  |  | 0.62 | *** | *** |
| *JAM2* | 0.49 | 0.30 | 0.78 | ** | * | -0.65 | *** | *** |
| *KIT* | 0.55 | 0.37 | 0.83 | ** | * | -0.68 | *** | *** |
| *LHFP* | 0.52 | 0.35 | 0.78 | *** | ** | -0.68 | *** | *** |
| *MS4A2* | 0.97 | 0.65 | 1.44 |  |  | -0.56 | *** | *** |
| *MYH11* | 0.64 | 0.45 | 0.90 | ** | * | -0.60 | *** | *** |
| *MYLK* | 0.53 | 0.36 | 0.76 | *** | ** | -0.64 | *** | *** |
| *PTTG1* | 1.72 | 1.06 | 2.80 | * |  | 0.69 | *** | *** |
| *TWIST2* | 0.72 | 0.49 | 1.07 |  |  | -0.51 | *** | *** |
| *TXNIP* | 0.89 | 0.56 | 1.41 |  |  | -0.55 | *** | *** |
| *TYMS* | 1.41 | 0.91 | 2.17 |  |  | 0.62 | *** | *** |
| *AKAP12* | 0.49 | 0.33 | 0.72 | *** | ** | -0.63 | *** | *** |
| *ALDH1A1* | 0.67 | 0.49 | 0.92 | ** | * | -0.66 | *** | *** |
| *ANLN* | 1.48 | 1.01 | 2.17 | * |  | 0.66 | *** | *** |
| *BIRC5* | 1.28 | 0.96 | 1.71 |  |  | 0.71 | *** | *** |
| *BTG2* | 0.94 | 0.66 | 1.33 |  |  | -0.52 | *** | *** |
| *C1S* | 0.63 | 0.45 | 0.90 | ** | * | -0.64 | *** | *** |
| *C7* | 0.64 | 0.47 | 0.87 | ** | * | -0.58 | *** | *** |
| *CCDC80* | 0.61 | 0.47 | 0.80 | *** | ** | -0.64 | *** | *** |
| *CCNB1* | 1.61 | 1.08 | 2.39 | ** |  | 0.66 | *** | *** |
| *CDC2* | 1.41 | 1.02 | 1.94 | * |  | 0.74 | *** | *** |
| *CDC20* | 1.38 | 0.90 | 2.13 |  |  | 0.62 | *** | *** |
| *CENPF* | 1.48 | 0.97 | 2.26 |  |  | 0.69 | *** | *** |
| *CEP55* | 1.71 | 1.16 | 2.53 | ** | * | 0.63 | *** | *** |
| *CTSG* | 0.77 | 0.57 | 1.03 |  |  | -0.53 | *** | *** |
| *FHL1* | 0.64 | 0.47 | 0.87 | ** | * | -0.60 | *** | *** |
| *ID4* | 0.73 | 0.57 | 0.94 | ** | * | -0.63 | *** | *** |
| *IGF1* | 0.61 | 0.45 | 0.85 | ** | * | -0.55 | *** | *** |
| *KIF23* | 1.59 | 1.05 | 2.40 | * |  | 0.66 | *** | *** |
| *MAPT* | 1.29 | 0.94 | 1.77 |  |  | 0.58 | *** | *** |
| *MKI67* | 1.32 | 0.92 | 1.88 |  |  | 0.68 | *** | *** |
| *MYBL2* | 1.79 | 1.15 | 2.79 | ** | * | 0.61 | *** | *** |
| *NGFR* | 0.53 | 0.37 | 0.75 | *** | ** | -0.53 | *** | *** |
| *OGN* | 0.70 | 0.54 | 0.90 | ** | * | -0.62 | *** | *** |
| *PDGFD* | 0.46 | 0.31 | 0.66 | *** | ** | -0.63 | *** | *** |
| *PKMYT1* | 1.42 | 1.05 | 1.93 | * |  | 0.68 | *** | *** |
| *RRM2* | 1.47 | 1.08 | 1.99 | ** | * | 0.74 | *** | *** |
| *SFRP1* | 0.68 | 0.52 | 0.89 | ** | * | -0.56 | *** | *** |
| *SMAD9* | 0.58 | 0.39 | 0.88 | ** | * | -0.63 | *** | *** |
| *TNXB* | 0.62 | 0.45 | 0.87 | ** | * | -0.55 | *** | *** |
| *TOP2A* | 1.24 | 0.96 | 1.61 |  |  | 0.72 | *** | *** |
| *TRIP13* | 1.66 | 1.01 | 2.72 | * |  | 0.60 | *** | *** |
| *TSPAN7* | 0.44 | 0.27 | 0.70 | *** | ** | -0.59 | *** | *** |
| *TWIST1* | 0.83 | 0.58 | 1.18 |  |  | -0.56 | *** | *** |
| *UBE2C* | 1.36 | 1.00 | 1.84 | * |  | 0.74 | *** | *** |
| *CD274* | 1.44 | 0.86 | 2.40 |  |  | -0.27 |  |  |
| *LAG3* | 2.23 | 1.27 | 3.95 | ** | * | -0.22 |  |  |
| *IDO1* | 1.06 | 0.60 | 1.88 |  |  | -0.20 |  |  |
| *CXCL9* | 1.08 | 0.80 | 1.47 |  |  | 0.09 |  |  |
| Durvalumab | 2.24 | 1.28 | 3.92 | ** | * | -0.20 |  |  |
| Immune.tolerance | 1.68 | 0.81 | 3.48 |  |  | -0.22 |  |  |

*** p<0.01, ** p<0.05, * p<0.1
